# Separation of ballistic and diffusive fluorescence photons in confocal Light-Sheet Microscopy of Arabidopsis roots

**DOI:** 10.1038/srep30378

**Published:** 2016-08-24

**Authors:** Tobias Meinert, Olaf Tietz, Klaus J. Palme, Alexander Rohrbach

**Affiliations:** 1 Laboratory for Bio- and Nano-Photonics, Department of Microsystems Engineering (IMTEK), University of Freiburg, Germany; 2 Institute for Biology II/Botany, Faculty of Biology, University of Freiburg, Germany; 3 BIOSS Centre for Biological Signalling Studies, University of Freiburg, Freiburg, Germany

## Abstract

Image quality in light-sheet fluorescence microscopy is strongly affected by the shape of the illuminating laser beam inside embryos, plants or tissue. While the phase of Gaussian or Bessel beams propagating through thousands of cells can be partly controlled holographically, the propagation of fluorescence light to the detector is difficult to control. With each scatter process a fluorescence photon loses information necessary for the image generation. Using Arabidopsis root tips we demonstrate that ballistic and diffusive fluorescence photons can be separated by analyzing the image spectra in each plane without a priori knowledge. We introduce a theoretical model allowing to extract typical scattering parameters of the biological material. This allows to attenuate image contributions from diffusive photons and to amplify the relevant image contributions from ballistic photons through a depth dependent deconvolution. In consequence, image contrast and resolution are significantly increased and scattering artefacts are minimized especially for Bessel beams with confocal line detection.

A fundamental principle in light microscopy is to uncover the composition of matter by sending photons with defined properties onto a piece of matter and detecting the scattered photons. However, things can become very complicated when the investigated object is large and of complex biological structures and multiple light scattering occurs. Photons that have been scattered too often lose their directional information, i.e. photons are no longer ballistic, but become diffusive. This loss of information does not only occur on the illumination side, but also on the detection side. When arriving on the camera, the origin of the photons is often unknown^1^. In consequence, an image becomes blurred and noisy.

A suitable microscopical method to tackle the problem of light scattering in both the illumination and detection path is light-sheet based microscopy (LSM)^2^,^3^. Besides advantages such as high acquisition speed, effective sectioning, high contrast and low phototoxicity, this technique is also fascinating because it allows to observe how the illumination light propagates from the side through selected planes of the object. Furthermore, the influence of the detection depth can be observed quite well. In this way, the analysis of both scattered laser light and fluorescence light helps to better understand the formation of useful image data and unwanted imaging artifacts^4^.

Many technical improvements have been achieved in LSM within the last decade - on the detection side, but especially on the illumination side^4^,^5^,^6^,^7^. It turned out that scanning a beam^8^ through the focal plane results in a more homogeneous light-sheet^4^ than by forming a static light-sheet generated by a cylindrical lens, known as selective plane illumination microscopy (SPIM). Besides the illumination with a scanned Gaussian beam, scanned Bessel beams offer intriguing advantages such as high penetration depths into media due to their self-reconstruction capability^5^. In addition, Bessel beams can generate more imaging contrast and resolution, when combined with confocal line detection^9^, two-photon excitation^10^ structured illumination^6^,^11^ or using coherent superposition to form a lattice light-sheet^12^.

Especially the use of beam shaping elements such as spatial light modulators (SLMs) or digital micromirror devices (DMDs) allows to generate nearly arbitrary illumination beams, which, in the optimal case, can be adapted specifically to the refractive index inhomogeneities of the object, which would otherwise lead to unwanted beam deflections and distortions^13^. This holographic shaping of the illumination beam requires coherent light, which is characterized by its unique phase dependency between all photons incident onto the object. However, fluorescence light emitted from the fluorophores inside the extended object lack this mutual phase dependency, since the emission of fluorophores is incoherent. Wavefront correction approaches on the detection side can help to improve the image quality^14^,^15^, but the significant computational efforts and several illumination iterations per image plane set strong limitations concerning the acquisition speed and hamper imaging of dynamic processes. Popular methods to improve the image quality by post-processing such as image deconvolution^16^,^17^,^18^ often remain unsatisfying because the point-spread function (PSF) at the camera is different for each position inside the 3D object, which is because fluorescence photons are scattered differently at each position. Nevertheless, post-processive deconvolution does not affect imaging speed.

The influence of scattering becomes apparent in an illustrative way when imaging large, scattering structures such as small plants and plant root tips, that consist of regularly shaped cells of variable sizes^19^, but reveal high refractive indices (high polarizabilities) leading to strong deviations of the illumination and detection photons. These effects degrade the image quality of those parts of the plant which are further away from the illumination lens and the detection lens. For example the small and dense pericycle cells within Arabidopsis roots appear blurry^19^. Lateral root initiation originates from the pericycle. Hormone gradients, which are established by polarity in the cell’s localized transport proteins of the PIN family, play a crucial role in this process^20^. Detailed real-time analysis of PIN proteins could not be performed so far because of limited sub cellular resolution and contrast. Although illumination from 4–5 sides helps to improve the image quality in the back parts of the object, this comes at the cost of a 4–5 -fold increase in illumination time and bleaching in addition to intensive postprocessing^2^.

Image formation in scattering media has been described by wave-optical models^21^,^22^ and investigated via Monte Carlo simulations^23^. An effective PSF has been introduced^24^,^25^ that allows image estimation^25^ and deconvolution^26^,^27^ under different scattering conditions. Image formation in scattering samples can be better understood by quantifying the amount of scattering events a photon has survived and considering this in specific PSFs^28^. However, a practical procedure to separate diffusive fluorescent photons or an analytical model describing their influence on the imaging process is still missing.

In this paper we introduce such a procedure and model. Based on the theoretical description of photon diffusion, we present a method that allows to separate efficiently the parts of the image resulting from the minimally scattered (ballistic) photons and the highly scattered (diffusive) photons. This information can be directly extracted from the image spectra such that no a priori knowledge about the object is required. We demonstrate the principle of separating weakly and strongly scattered fluorescence photons using identical Arabidopsis Thaliana roots, which we image in four different illumination and detection modes. We have developed a concept that quantifies the change of ballistic photons into diffusive photons, which varies with the detection depth and which is implemented in a depth dependent deconvolution of the 3-D images.

## The Conceptual Approach. 

In this study we investigate the influence of three important components of the imaging chain - the illumination beam, the detection method and the image postprocessing - on the 3D image quality. Further, this chain is studied in four different imaging modes. All variations of these components are performed at exactly the same biological object, an Arabidopsis thaliana root, to enable a meaningful comparison of the imaging modes. The roots are about 120 μm in diameter and scatter strongly both the illumination light and the fluorescent detection light, leading to reduced contrast and resolution, but also to local image artifacts in the back parts of the object.

**Figure 1:**
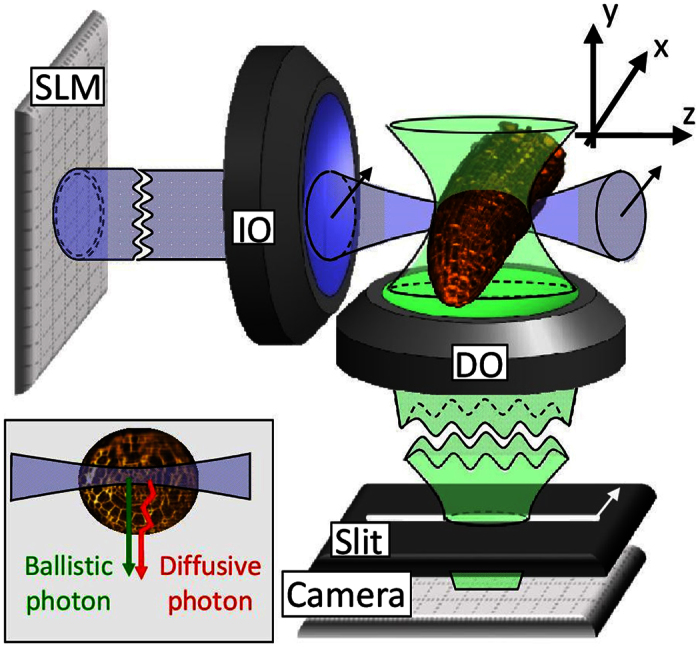
Sketch of the microscope. A spatial light modulator (SLM) in the illumination path allows switching between Gaussian and Bessel beam illumination for comparative measurements. Confocal line detection is possible through a synchronized movement of the illumination beam with the rolling slit of the camera, thereby increasing image contrast and resolution. DO: detection objective, IO: illumination objective. Inset: Scheme for the emission of ballistic and diffusive photons in light-sheet microscopy.

As the first component of the imaging chain, we use a spatial light modulator (SLM) that can switch plane wise the shape of the illumination beam, which is either a Bessel beam or a Gaussian beam optimized to the extent of the object. The beam is scanned laterally in the plane of focus (see Scheme of Fig. 1). As the second component, we use a camera with a rolling shutter, which enables confocal line detection^9^,^29^ by a narrow width of the slit moving parallel to the illumination beam (see Fig. 1) or conventional widefield detection for a large slit width.

The third component in the imaging chain is a deconvolution based on our model describing the photon diffusion in image formation. The required scattering parameters can be extracted by analyzing the width of the image spectra, which decrease with the detection depth and thereby represent the increase of image blur. We will show that enhancing high frequency components by deconvolution enhances those image components, which mainly originate from ballistic photons. This way, the object and depth dependent deconvolution can be interpreted as post-processive photon separation.

## Results

### 3D imaging of root tips

Arabidopsis thaliana root tip is the preferred model for root development in plants due to its small size and simplicity. With conventional light-sheet microscopy only outer, but not inner cell layers were accessible for microscopic analysis with reasonable resolution^19^.

Here, we imaged Arabidopsis thaliana roots expressing the plasma membrane located protein LTi6b fused to GFP homogenously in all cells^30^. Seeds of 35s::LTi6b-eGFP) were germinated for 7 days on solid medium in the light. Excised roots were embedded in 1.5% low-melting agarose.

It is well known that Gaussian and Bessel beams exhibit different scattering properties in inhomogeneous media and thereby generate different light-sheets and images of different quality in LSM. The usage of a spatial light modulator (SLM) in the illumination path allows to shape nearly arbitrary illumination beams, which will then be scanned laterally across the object as indicated in Fig. 1. This offers the opportunity to directly compare the images acquired by using e.g. Gaussian or Bessel beam illumination^31^.

The concentric ring system around the thin main lobe of a Bessel beam enables the superior propagation stability and the self-reconstruction capability of the beam. However, it is also the ring system that generates the low image contrast because it excites fluorescence also out of the focal plane. This problem can be well solved by using a confocal line detection scheme, which mainly detects the fluorescence generated by the thin main lobe of the Bessel beam and generates unreached contrast and resolution^9^. Synchronizing the light-sheet mode of the camera (ORCA-Flash4.0, Hamamatsu) with the scanned illumination beam allows confocal line detection at high framerates^29^ and excellent sectioning. In this way 3D image stacks including image aberrations can be obtained with high sampling also in a plane vertical to the light-sheet revealing so far unknown imaging peculiarities on a sub-micron scale.

**Figure 2:**
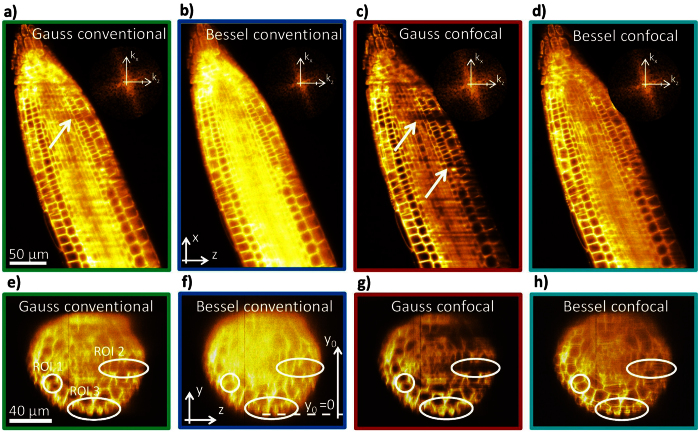
Image cross-sections from Arabidopsis root tip using 4 different imaging modes. (**a–d**) Slices through images of Arabidopsis root along illumination(z)- and scanning(x)-direction. Image contrast is clearly enhanced by confocal detection for both Gaussian and Bessel beam illumination. Whereas the Bessel beam can self-reconstruct in inhomogeneous media, the Gaussian beam cannot and generates stripe artefacts. However the Gaussian beam reveals the best contrast, also revealed by the image spectra in the upper right corner of each image. (**e–h**) Image slices along the illumination(z)- and detection(y)-direction. Beside the stripe like artefacts (ROI 2), Gaussian illumination shows aberrations at certain structures, such that horizontal cell membranes disappear completely (ROI1 and 3). This indicates strong scattering of a Gaussian illumination beam while propagating in the cell membrane.

In total four 3D image stacks each consisting of 260 planes separated by Δy = 0.5 μm were recorded from a single root tip. To allow a fair and precise comparison without bleaching or drift effects, each plane was imaged successively with the above mentioned four different imaging modes. Both the Gaussian (axial full width at half maximum (FWHM) Δz = 300 μm) and the Bessel beam (axial FWHM Δz = 300 μm; numerical aperture NA = 0.15) illumination have been used to image in conventional widefield mode (detection slit width *d*_slit_ = *M*_*T*_ · 50 μm) and in confocal line detection mode (detection slit width *d*_slit_ = 8 · 6.5 μm = *M*_*T*_ · 1.3 μm corresponding to 8 pixels on the camera, at magnification *M*_*T*_ = 40). Figure 2a,b show image slices of an Arabidopsis root tip parallel to the light-sheet with Gaussian and Bessel illumination and conventional detection. Whereas the Gaussian beam reveals the better image contrast, the Bessel beam image hardly shows artifacts caused by scattering of the illumination light as visible with the Gaussian beam marked with a white arrow. In the top right corner the Fourier transforms of the images are displayed (in the spatial frequency coordinates k_x_ and k_y_) revealing a broader image spectrum (better transfer of high frequencies) for the conventional Gaussian beam.

The combination with confocal line detection results in a distinct contrast improvement for both the Gaussian beam and the Bessel beam as can be seen in Fig. 2c,d and also in the broadened image spectra. Although the image contrast is still better with the Gaussian beam, the scattering artifacts in the image become more pronounced especially in the back part of the image, since the Gaussian beams are stronger deflected and scattered than the Bessel beams^9^ and thereby do not propagate straight along the detection slit of the camera. In consequence, significantly less ballistic photons are collected and the image reveals dark stripes. On the other side the image obtained with Bessel beam illumination combined with confocal line detection reveals a good contrast with much less artifacts since fluorescence from the ring system is efficiently blocked.

For a complete comparison of the beam propagation and imaging properties of all four imaging modes image slices perpendicular to the light-sheet have to be inspected. Figure 2e–h show examples of such yz-slices giving emphasis to three regions of interest (ROI). Again stripe-like artefacts are visible using Gaussian illumination (ROI2). However, at closer look areas with horizontal structures (ROI1 & ROI3) reveal additional significant artefacts in the case of Gaussian illumination. The bright, mostly regular structures in the images indicate fluorescently labeled cell membranes being hit by illumination photons. However, missing horizontal connections between cells point out that illumination photons from the Gaussian beams do not reach several horizontally oriented membranes because of strong scattering of the laser light. Because of the conical propagation direction of the photons in the Bessel beam, also structures parallel to the light-sheet are imaged with good contrast (see also Supplementary Text 1).

### Depth dependent image contrast

**Figure 3:**
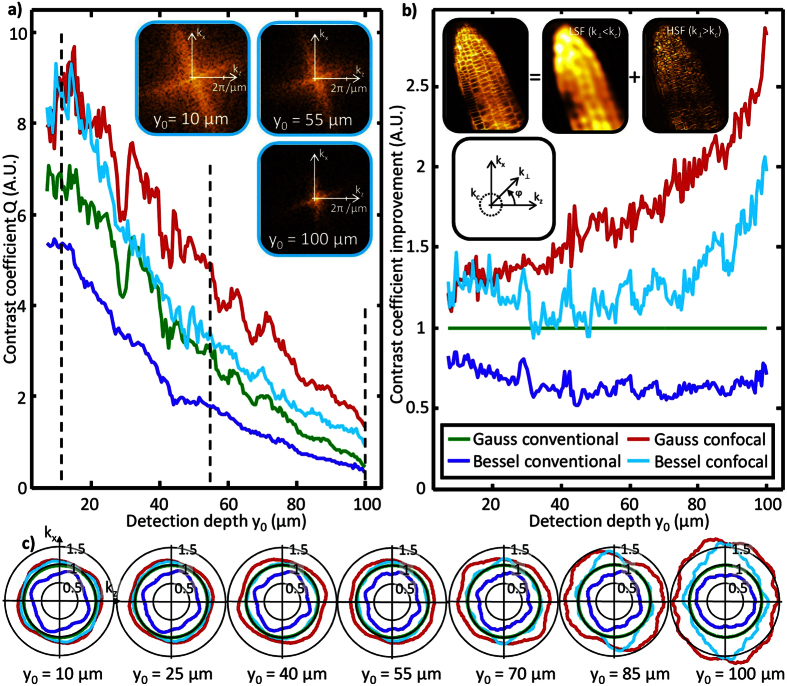
Depth dependent image contrast for different illumination and detection modes. (**a**) Quantitative comparison of image contrast by the contrast coefficient *Q(y*_*0*_). The contrast decays nearly exponentially for increasing detection depth *y*_*0*_. The 3 insets show image spectra at different detection depths for Bessel illumination and confocal detection. The spectra become narrower for higher detection depths. (**b**) The contrast coefficient is normalized to that of the standard Gaussian illumination with conventional detection. An enhanced contrast improvement by confocal detection is observed for high detection depths. The first inset illustrates the separation of useful high spatial frequency (HSF) information and low spatial frequency (LSF) background in Fourier space. The ratio of the mean value of the HSF and LSF components in Fourier domain gives the contrast coefficient. The second inset shows the relevant coordinate definitions in Fourier space. (**c**) Width of image spectra from 2D slices at different detection depths normalized to the Gaussian illumination with conventional detection. The 1/e width in all directions is obtained by exponential fitting in all directions. The elliptical shape of the confocal spectra for high detection depths indicates the contrast improvement perpendicular to the detection slit (x-direction).

It was shown by the yz-slices of Fig. 2 that for all imaging modes a strong decrease in image contrast is going along with an increasing detection depth (y-axis). One method to provide a quantitative measure of contrast is the “useful contrast” introduced by Truong *et al.*^32^. A similar procedure is applied in our study and is further described in the methods section. This approach determines the contrast coefficient *Q*, which quantifies the ratio of the high spatial frequency image components (HSF, *k*_*⊥*_ > *k*_*c*_) and the low spatial frequency image components (LSF, *k*_*⊥*_ ≤ *k*_*c*_) (see eq. (11)). The corner-frequency *k*_*c*_ = 2π/*d*_*cell*_ used for the spatial filtering is defined by the largest object structure, which is the longer cell length of *d*_*cell*_ ≈ 20 μm (see inset of Fig. 3b).

Therefore, a high value of *Q* refers to high contrast in an xz-image slice. The contrast coefficient is plotted in Fig. 3a for all imaging modes over the detection depth *y*_*0*_ (*y*_*0*_ is defined in Fig. 2f). As expected, a strong decrease in contrast is visible with increasing detection depth. The drop in contrast *Q* caused by a few 10 micrometers propagation through the object is even stronger than the contrast difference caused by the imaging mode. This effect points out that the compensation for the detection depth induced loss in contrast is of great importance and might be differently effective for each imaging mode.

For a quantitative comparison of the different imaging modes, the contrast is normalized by the contrast coefficient of the standard imaging mode (Gaussian illumination and conventional detection). The result is plotted in Fig. 3b. It is obvious that confocal line detection improves the contrast. At low detection depth (*y*_*0*_ = 10 μm) the contrast is improved roughly by 70% for Bessel illumination (compare the bright and dark blue lines) and by 30% for Gaussian illumination (the red line above the green line). The contrast improvement is further enhanced for higher detection depths. For e.g. *y*_*0*_ = 100 μm the contrast coefficient is improved by more than 150% for both illumination modes. For higher detection depth the amount of diffusive photons involved in the image formation increases and image quality drops down. Since diffusive photons are multiply scattered on the way to the detector and thus are displaced in the image plane, they have a higher probability to be blocked by the confocal slit. This effect leads to the enhanced contrast improvement by confocal detection for higher detection depths.

Since the slit is parallel to the z-axis, only photons displaced in x-direction are blocked thereby increasing optical resolution along x. This effect is shown in Fig. 3c, where the 1/e width of the spectra in all directions of the k_x_k_z_-plane are plotted relative to the spectrum obtained by the standard imaging mode (see the green circle as a reference). The elongation of the confocal image spectra in k_x_-direction demonstrates that the improved contrast enhancement at high detection depths is caused by the confocal slit, which works in in x-, but not in z-direction. A detailed discussion on the effect of confocal detection with special attention on the difference between Bessel and Gaussian illumination can be found in Supplementary Text 2.

### Image formation with ballistic and diffusive photons

The image is formed by ballistic (hardly scattered) and diffusive (multiply scattered) photons. Due to the fact that diffusive photons carry only little high-frequency information about the object, the image quality decreases if the amount of diffusive photons involved in the imaging process increases. Consequently, the image quality is increased if the percentage of ballistic photons is enhanced. This process is called gating^33^,^34^. Confocal detection is a first step in this direction since the detection slit blocks photons, which are displaced by multiple scattering. A more advanced method is separating ballistic and diffusive photons by their time of flight^35^,^36^ but technical complexity has prevented a combination with LSM. However, an alternative, which needs no further hardware, is separating the effect of the ballistic and diffusive photons on the image in a post processing step. The defined suppression of low-frequency information from the diffusive photons and the defined enhancement of high-frequency information from the ballistic photons is nothing else than a deconvolution with a PSF describing both the optical response and the detection depth dependent object response.

A diffusive photon differs from a ballistic photon by its propagation angle θ. Any angular change during the propagation of a fluorescence photon on its way to the detector will result in a wrong position on the camera and to a wrong image contribution. The image is described by 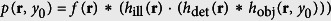, where *h*_ill_(**r**) and *h*_det_(**r**) are the purely optical response functions for light-sheet illumination and detection. (Further explanation of the mathematical description of the image process can be found in the methods section and in Supplementary Text 6) The contribution from both the ballistic and diffusive photons is defined by the scattering properties of the object *f*(**r**) and can be accounted for quantitatively by an object response function *h*_obj_(**r**, *y*_0_), which consists of two parts. One part for diffusive photons, which is nearly of Gaussian shape and broadens with the detection depth y_0_ and one part describing the ballistic photons by a delta-point function. This is explained in detail in the methods section. The degree of the linearly increasing influence of the object, i.e. the broadening of *h*_obj_(**r**, *y*_0_) is expressed by the scattering parameter *γ*, which is a material constant specific for the object. The corresponding object transfer function *H*_obj_(**k**_**r**_, *y*_0_), i.e. the Fourier transform of the response function, describes the signal loss for each spatial frequency **k**_**r**_. It consists of a Gaussian like part for the diffusive photons that narrows with *y*_*0*_ and a constant offset for the ballistic photons that drops off exponentially with *y*_*0*_:


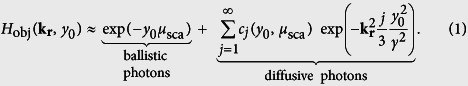


**Figure 4:**
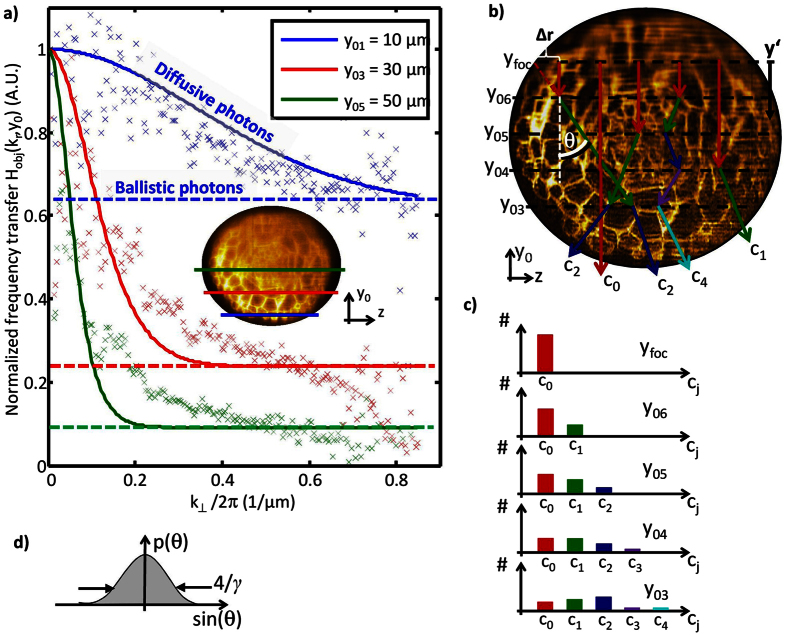
Photon diffusion through scattering object and its effect on the frequency transfer. (**a**) Averaged object transfer function *H*_obj_(*k*_⊥_, *y*_0_) for three different detection depths y_0_. Photon scattering leads to a suppression of higher spatial frequencies for larger y_0_. *H*_obj_(*k*_⊥_, *y*_0_) is composed of a Gaussian like function representing the frequency filter for scattered photons and a constant part given by the fraction of ballistic photons. Model based fitting to the processed image data enables the extraction of scattering parameters. (**b**) Scheme for photon diffusion through the object. While some photons leave the object unscattered (fraction c_0_), others undergo n scattering events (fraction *c*_*n*_). Each scattering event results in a random change of the propagation direction *θ*. Since the propagation angle changes in a layer (*y*_01_–*y*_06_) out of the focal plane, the origin of emission appears to be displaced by *Δr* in the image thus leading to blurring images. In a first approximation the effect increases linear with the defocus y’. (**c**) Subdivision of photons into photon fractions of different scattering order for different detection depths *y*_0_. (**d**) The probability to change the propagation direction by *θ* is assumed to follow a Gaussian distribution p(*θ*), which is characterized by the material parameter γ.

The shape and behavior of the object transfer function *H*_obj_(*k*_⊥_, *y*_0_) in lateral direction is shown in Fig. 4a for three different detection depths *y*_*0*_. The experimental data is obtained through angular averaging of the normalized image spectra. Figure 4b,c illustrate photon propagation through the object, where a small fraction *c*_*0*_ of the fluorescent photons leaves the object unscattered, and fractions *c*_*j*_ are scattered *j* times. The parameter *μ*_sca_ describes the probability that a photon is scattered. Each scattering event results in a random change of the propagation direction θ. The probability for a directional change follows a Gaussian distribution p(θ), characterized by the material parameter *γ* (see Fig. 4d). In detail explanation of eq. (1) can be found in the method section.

### Depth dependent frequency transfer

The image spectra shown as insets in Fig. 3a for y_0_ = 10 μm, 55 μm and 100 μm reveal that the spectral widths (xz-slices) decrease for higher detection depths. This means that a reduced amount of high frequency information is transferred through the scattering sample. To extract this loss in high frequencies caused by the object, the spectra must be normalized by a reference spectrum at the bottom of the object (see eq. (12)), where the influence of the object is assumed to be negligibly small. Division by this reference spectrum eliminates the effect of the microscope and the frequency spectrum of the object. Hereby we assume that the object frequencies do not change significantly with the detection depth. Deviations from this assumption are elaborated in the discussion. The obtained frequency transfer is plotted as scattered markers for three different detection depths (y_0_ = 10 μm, 30 μm and 50 μm in Fig. 4b). The data has been extracted from the 3D image data captured with Gaussian illumination and conventional detection. The frequency transfer is independent on the direction and can be plotted over the radial spatial frequency 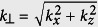. As expected from theory (see method section), the frequency transfer consists of a Gaussian like part centered around *k*_⊥_ = 0 describing the diffusive photons and a constant frequency transfer. Since ballistic photons are not affected by the object, their spatial frequencies do not change, which results in a constant frequency transfer function. The width of the Gaussian part reduces with increasing detection depth *y*_*0*_ and the constant level drops exponentially with y_0_ as expected from Lambert-Beer’s law.

Extracting the object transfer function allows to compensate for the reduced high frequency transfer through the object by applying a deconvolution according to our model for an effective system PSF. Therefore, the scattering parameters *μ*_sca_ and *γ* which describe the scattering behavior averaged over the 3D image have to be known. If no such parameters are available for example for heterogeneous materials like Arabidopsis roots the parameters can be extracted from the images. This procedure is described in the methods part and in Supplementary Text 3. By fitting *H*_obj_(*k*_⊥_, *y*_0_) to the frequency transfer data, we found *μ*_sca_ = 50 mm^−1^ and *γ* = 22.4. The model-fitted curve is plotted in Fig. 4a with solid lines. Supplementary Movie 1 shows the fitting result over the whole range of the detection depth. As mentioned above, the frequency transfer has been calculated for the conventional detection mode since it is independent of the direction in the k_x_k_z_-plane. This is different for confocal detection. Nevertheless, it is possible to extract the scattering parameters by only considering the frequency transfer in k_z_-direction (See Supplementary Text 3 for more details).

### Depth dependent deconvolution

With knowledge of the scattering parameters *μ*_sca_ and *γ*, a depth dependent deconvolution is possible. Here, we used a Wiener filter (see methods section) instead of more advanced deconvolution algorithms, to make the effect of photon separation better visible. The deconvolution intensifies the influence of ballistic photons on the imaging process at high detection depths, such that it can be classified as a gating technique.

**Figure 5:**
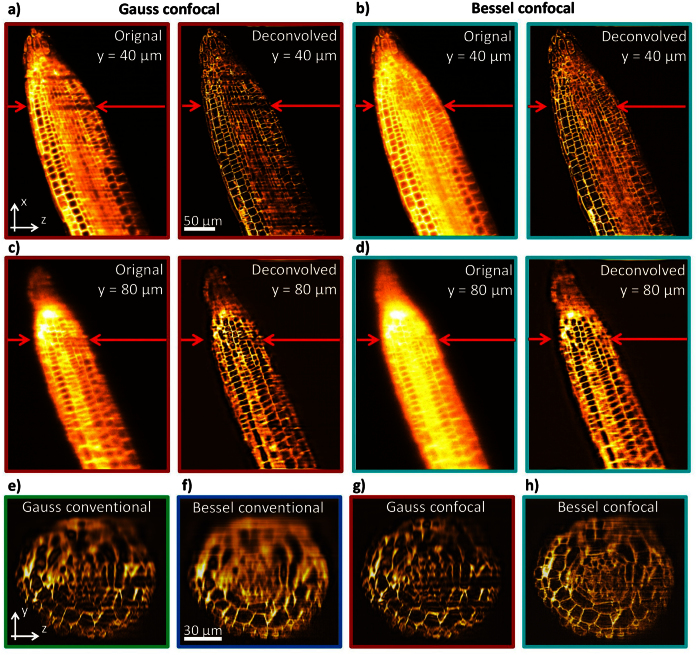
Image cross-sections of a 90 μm thick Arabidopsis root tip with confocal detection without and with depth dependent 3D deconvolution. (**a–d**) xz-slices comparing the effect of a depth dependent deconvolution for two illumination modes at a detection depths of y_0_ = 40 μm and 80 μm. (**e–h**) yz-slices of depth adapted deconvolved images with different illumination and detection modes. A strong contrast enhancement is evident relative to Fig. 2. Further aberrations in horizontal structures become visible for Gaussian illumination. The x-position of the yz-slices are marked by red arrows in (**a–d**).

Figure 5a–d show how contrast of xz-slices is increased by a depth-dependent deconvolution. It displays how the enhancement of high frequencies is adapted to the blur which increases with the detection depth. Figure 5e–h show yz-slices through 3D stacks, which have been processed by a depth-dependent deconvolution. 12 different y_0_-position and 12 different PSFs (see methods section) have been used. The images reveal a strong increase of contrast compared to the unprocessed images displayed in Fig. 2e–h.

Some overshooting is visible in the left side of Fig. 5h. This is due to the fact that the round shape of the root is not considered and the effective detection depth in the area, where overshooting appears, is shorter than expected. Additional scattering of the illumination light, which is not considered, leads to a widening of the illumination beam from left to right, causing a gradient of image quality. Compensating for the scattering of illumination light needs a separate discussion, because of the coherent nature of the illumination beam.

## Discussion

### 3D imaging of root tips with and without confocal detection

The advantages of confocal line detection using a camera with a moving slit (rolling shutter) become clearly visible in Fig. 2. The image contrast improves, the lateral image spectra become broader (see insets) and especially the resolution in detection direction is improved (Fig. 2g,h). However, this comes at the cost of stronger stripe artifacts for Gaussian beam illumination, which do not exhibit the directional stability of Bessel beams. Gaussian beams are deflected during propagation and after some distance do not propagate parallel to the detection slit. In addition, it becomes apparent by the yz-slices of Fig. 2 that Gaussian beams cannot illuminate entirely the cell membranes oriented parallel to their propagation direction. However, this effect is not further investigated here. Bessel beams on the other side provide good contrast over the extent of the 100 μm thick root without producing any significant artifacts, which is the consequence of their propagation stability and their self-reconstruction capability.

A recent study using structured light-sheet illumination reveals a strong image darkening in the central part of the root tips, since the illumination modulation frequency, does not match the PSF broadening. In our study, however, the small central cells can be reasonably resolved^19^. In structured illumination, the frequency of the illumination pattern has to be adapted to the PSF width and thereby also to the detection depth in order to exploit its full power, which would be possible with our model.

The gating effect of a confocal slit, i.e. the blocking of diffusive photons, has been analyzed also theoretically. It was shown by Supplementary Figs 3 and 4 that contrast improvement by confocal detection using Gaussian illumination is mainly caused by blocking diffusive photons, but not by blocking fluorescence excited out of the focal plane. In other words, confocal detection in weakly scattering media with Gaussian illumination has almost no effect. In contrast Bessel illumination with confocal line detection benefits from the true confocal effect and gating further improves contrast.

### Separating fluorescence photons in the image

Ideal, diffraction limited imaging is only possible by ballistic photons, which transfer information from the focal plane inside the object to the detector. Since the number of ballistic photons at the detector decreases exponentially with the detection depth y_0_, the useful image signal decays in the same way. The image background, defined by the diffusive photons, will increase exponentially with the detection depth. The separation between ballistic and diffusive photons, i.e. a strong enhancement in contrast, is possible, as long as the amount of ballistic photons is distinguishable from the noise level.

To extract the frequency transfer through the object each image spectrum at y_0_ is divided by the image spectrum at y_0_ ≈ 0, which is mainly defined by the imaging optics. In principle, the normalized spectrum can be obtained experimentally without the fit functions derived in our model. However, the fit functions provide an additional control and deliver the material parameters *μ*_sca_ and *γ*.

Figure 4 summarizes the change of the object transfer function for different detection depths *y*_*0*_. Both the experimentally and theoretically obtained *H*_obj_(**k**_**r**_, *y*_0_) reveal a quite good coincidence in shape and thereby demonstrate the ongoing transfer of ballistic photons to diffusive photons with increasing *y*_*0*_. Deviations between the measurement data and fit curves can be first a consequence of the inhomogeneous scattering properties of the root tip and second, the characteristic structure size and thus the object spectrum changes with detection depth, such that the effect of the object spectrum cannot be completely eliminated by scaling with a reference spectrum. For example, cells in the middle of the root (Endodermis, Pericycle and Stele) have a size of 5 to 7 μm and are smaller than the cells in the two outer cell layers of the root (Epidermis and Protoxylem), which measure 10 to 20 μm. Since the reference spectrum was obtained from an outer cell layer, frequencies from 0.05 to 0.1 μm^−1^ are more pronounced than in a layer at 50 μm detection depth (green curve in Fig. 2b)). Thus, in the normalized spectrum at *y*_*0*_ = 50 μm frequencies from 0.05 to 0.1 μm^−1^ are reduced and frequencies from 0.14 to 0.2 μm^−1^ occur amplified. The fitting algorithm turned out to be robust against these deviations. In practice, there are several objects offering depth independent object spectra (e.g. Zebrafish with labeled nuclei) and thus are even more suitable for the presented method. Nevertheless, by imaging membrane labeled root tips, we demonstrate that the method is not limited to such samples.

### Depth dependent deconvolution

Deconvolution with a single response function generates images with either too little or too much contrast (effect of overshooting) depending on the choice of the PSF. However, by adapting the object PSF to the corresponding detection depth, resolution and contrast in the image can be optimized over the complete 3D volume. For illustration of this effect, see Supplementary Text 4. The depth dependent deconvolution algorithm was tested successfully also at a dense bead cluster. Results are shown and discussed in Supplementary Text 5.

Our results prove that scattering of fluorescent light can be well approximated by a PSF even for inhomogeneous samples like Arabidopsis roots. In comparison to other deconvolution techniques, which utilize point like structures in the object to extract the PSFs^37^ our technique can be applied to any structure without inserting beads. Recently developed blind deconvolution algorithms are based on a depth-variant PSF model^38^. Here, spherical aberrations from refractive index steps between immersion medium and sample were considered multiple scattering a main source for image degradation is neglected.

A perfect depth-dependent deconvolution would require a complete 3D deconvolution for all N xz-planes. A solution with less computational effort is described in the methods part, where twelve 3D deconvolutions for the whole stack have been performed. However, it is not necessary to compute twelve 3D deconvolutions for the whole stack, since only a limited number of planes around the specific plane at *y*_*0*_ is of interest. A further reduction of computational efforts is possible, if each of the twelve 3D deconvolution is performed on a section, which is six times thinner than the whole stack. In the end, the computational effort will increase only by a factor of two relative to a standard 3D deconvolution with a single PSF.

Furthermore, all image processing operations can be outsourced directly to camera systems exploiting fast and flexible power of GPUs or FPGAs.

By the strong increase in contrast it becomes apparent that Bessel illumination with confocal detection benefits most from the deconvolution and leads to a nearly artefact-free image. The already mentioned artefacts caused by Gaussian illumination are even more obvious after deconvolution. Only without deconvolution, one may argue whether the high contrast achieved by Gaussian illumination or the elimination of artefacts by Bessel illumination leads to the best image quality. In general, this question has to be answered individually for each application. After deconvolution the best image quality is clearly achieved by Bessel illumination.

### Extracting scattering parameters out of the image

It was shown in Fig. 4a that the model-based transfer function 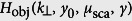 introduced in eq. (1) fits reasonably well to the frequency transfer extracted from the experimental data. The decrease of the offset level allowed to extract *μ*_sca_, whereas the decrease in Gaussian widths allowed to extract *γ*. We tested the universality of our approach by using the two different illumination and detection modes, to extract the same material parameters of the same root tip. Because of the gating effect by confocal detection the influence of the various scattering orders on image formation is changed. Thus the weighting by the photon fractions in the superposition of eq. (1) has to be modified in order to enable the fitting of the frequency transfer extracted from confocal images.

Alternative methods like collimated transmission measurements and goniometry^39^ require thin samples. Our model is also applicable to thick samples, which can be described neither by the quasi ballistic regime nor by the diffusive regime^40^.

The results are summarized in the following table (Table 1) and indicate that the extracted material parameters *μ*_sca_ and *γ* agree with each other reasonably. Further details and illustrating movies can be found in the Supplementary Text 3. In Supplementary Text 5, the scattering parameters of a bead cluster were extracted. It was shown that the scattering parameters are close to the values predicted by Mie theory.

It will be interesting in biology to analyze both scattering parameters over a longer time, e.g. during root tip development and during responses to external stimuli, since they provide biophysical, structural and possibly mechanical information about the state of the cellular compound. This information is usually not visible with standard fluorescence imaging. It will be an interesting future task to extract this information spatially resolved over areas of different cellular morphology within the root tip.

## Conclusion

A severe and unsolved problem is the multiple scattering of fluorescent light on the way to the detector, thereby blurring the images. This effect is the stronger, the larger the object and the longer the way of the photons through the scattering object. We have presented an effective and elegant solution to this problem, leading to a significant improvement in image quality. We separated the image contributions of the diffusive and the ballistic photons by postprocessing. Our approach, which does not require a priori information, neither slows down the acquisition speed of light sheet microscopy, nor are any iterative image acquisitions required, as this is the case with adaptive optical approaches. Our algorithm is applicable to most new and existing 3D data sets suffering from image blur due to scattering of fluorescence light. It should be beneficial to every light-sheet microscopist and could in principle be applied to other microscopy techniques.

## Methods

### Theory of image formation

#### Image generation in light-sheet microscopy

The formation of a 3D image *p*(**r**) can be described by a convolution (symbol *) of a 3D object distribution *f*(**r**), e.g. the fluorescence emission distribution, with the system PSF *h*_sys_(**r**):


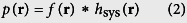


The impulse response *h*_sys_(**r**) of the imaging system is assumed to be shift invariant and contains the information on resolution and contrast. In LSM an effective systems-PSF *h*_sys_(**r**) can be defined, which is given by


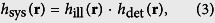


where *h*_ill_(**r**) and *h*_det_(**r**) are the effective illumination PSF and the detection PSF respectively^9^,^41^. The light-sheet is formed by a single beam with intensity *h*_SB_(**r**) scanned with velocity 
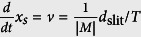
, with *T* being the exposure time of the camera and *d*_*slit*_ the slit width, if confocal detection is applied. For better readability the transversal magnification of the detection optics is set to |*M*| = 1. Thus the illumination PSF effective for one pixel line during T reads


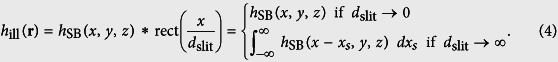


The influence of the rectangular slit function 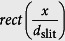 is effective through a convolution in x-direction with the radially symmetric beam *h*_SB_(**r**) (see further explanations in the Supplementary Text 6). In the case of conventional widefield detection, the slit width is very broad, such that *d*_slit_ → ∞ and 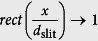. Thereby the light-sheet intensity 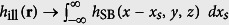 is maximally broad in x-direction. However, in the case of confocal line detection the slit width is very narrow, such that *d*_slit_ → ∞ and 
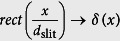
 and thereby *h*_ill_(**r**) → *h*_SB_(**r**). In this case the system PSF is 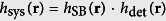, resulting in a confocal PSF in x- and y- directions. Since the illumination NA is smaller than the detection NA and thus *h*_ill_(**r**)is much broader than *h*_det_(**r**), the multiplication 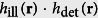 is mainly effective in detection direction y, i.e. normal to the light-sheet. It should be emphasized that the confocal PSF 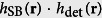 is mainly beneficial if the profile of *h*_ill_(*y*) in y-direction is broadened compared to *h*_SB_(**r**). This is the case for a Bessel beam with lateral profile 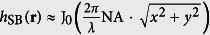, but not for a Gaussian beam. Therefore confocal detection leads to a strong suppression of the fluorescent background made for Bessel beam illumination^9^.

#### Image generation in combination with an object response function

If LSM is used to capture a 3D image stack of strongly scattering objects a significant decrease in image quality with increasing detection depth *y*_*0*_ can be seen. This loss of image quality with *y*_*0*_ corresponds to a low pass filtering, which can be modeled by a convolution with an additional object response function *h*_obj_(**r**, *y*_0_) describing the influence of the object on the detection process. Consequently, *h*_det_(**r**) is replaced by *h*_det_(**r**) * *h*_obj_(**r**, *y*_0_) and the effective system PSF can be written as


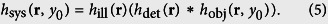


The fact that *h*_sys_ is now a function of *y*_*0*_ indicates that the image process is no longer a 3D shift invariant problem and *h*_sys_(**r**, *y*_0_) should be used for one specific detection depth *y*_*0*_. Thus, the final 2D-image obtained from position y_0_ is given by


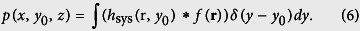


Eq. (5) shows that the influence of *h*_obj_(**r**, *y*_0_) on the image process is highly anisotropic and depends on *h*_sheet_(**r**). A good light-sheet has only a small extent in detection direction y. Due to the multiplication in eq. (5), the spread caused by *h*_obj_(**r**, *y*_0_) is limited to the light-sheet thickness. The same is true in scanning direction x, if confocal detection is applied (*d*_slit_ → 0, *h*_ill_(**r**) → *h*_SB_(**r**)). This results in a gating effect.

#### Depth specific scattering of fluorescence photons

To understand the depth dependence of *h*_obj_(**r**, *y*_0_), one has to consider the exponential decrease of the number of ballistic photons and the increase in diffusive photons that travel over the distance *y*_*0*_ through the object towards the detector. The larger the detection depth *y*_*0*_, the larger is the average of the scattering order *j*, which indicates the number of scattering events a fluorescence photon involved in the imaging process has survived. In this way, *c*_*j*_(*y*_0_) denotes the percentage of *j* times scattered photons at depth *y*_*0*_, such that *c*_*j*=0_(*y*_0_) is the fraction of ballistic photons contributing to the image. Since every scattering order carries a different amount of information, the image process for each order *j* is described by a specific object response *h*_obj,*j*_(**r**, *y*_0_). Then the overall object response *h*_obj_(**r**, *y*_0_) is given by the weighted superposition


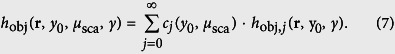


As indicated by eq. (7), the fraction *c*_*j*_ depends on the scattering coefficient *μ*_sca_, which describes the probability for a photon to be scattered while propagating a certain distance through the object. By neglecting absorption, one finds (see Supplementary Text 7)


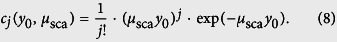


**Figure 6:**
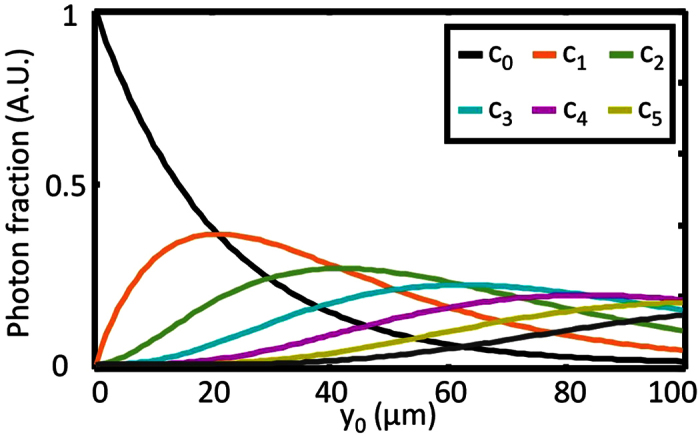
Photon emission from different detection depths inside the scattering object. Composition of all emitted photons by groups of photons of different scattering order. With increasing detection depth the fraction of ballistic photons (*c*_*0*_) drops exponentially (black curve), while one (*c*_*1*_) and multiple times (*c*_*n*_) scattered photons gain influence.

For ballistic photons (with *j* = 0), *c*_*j*_(*y*_0_) results in the Lambert-Beer law, exp(−*μ*_sca_*y*_0_). The term 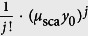 extends the Lambert-Beer law to higher scattering orders. Figure 6 shows an example of the distribution of fluorescent photons over the scattering orders given by eq. (8).

The second term in eq. (7) describes the change in shape of the specific object response *h*_obj,*j*_(**r**, *y*_0_), which depends on the scattering order and can be approximated by


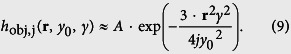


where *A* is an unimportant normalization factor. A detailed derivation for eq. (9) can be found in Supplementary Text 8. The broader the specific object response function, the more high-frequency information is lost. This loss in information increases with every scattering event and with the detection depth *y*_*0*_ to the focal plane. It is important to note that for ballistic photons *h*_obj,*j*=0_(**r**, *y*_0_) becomes a δ-function. This is in agreement with the fact that these photons are not affected by the object. *h*_obj,*j*_(**r**, *y*_0_) also depends on the material specific unit *γ*, which describes the angular width of the scattering phase function 
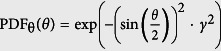
, with *θ* being the scattering angle (see Fig. 4b,d)). The relation to the anisotropy factor *g*_HG_, known from e.g. the Henyey-Greenstein function^42^, is given by 
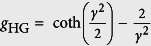
 with coth being the hyperbolic cotangent. We find *γ* → 0 for Rayleigh scatterers (PDF_θ_(*θ*) = 1) and *γ* → ∞ for forward scatterers (PDF_θ_(*θ*) = *δ*(*θ*)).

In Fourier domain one finds the depth dependent fluorescence photon object transfer function *H*_obj_(**k**_**r**_, *y*_0_) 
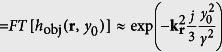
, which describes the influence of the object only (hereby no prefactor is needed; see Supplementary Text 8). Since *h*_obj_(**r**, *y*_0_) was assumed to be a sum of Gaussian functions (see eq. (7)), the Fourier transform *H*_obj_(**k**_**r**_, *y*_0_) 
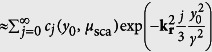
 also consists of a sum of Gaussians. The contribution of the ballistic photons in real space is described by an infinitely small Gaussian function identical to a Dirac delta function. Accordingly in Fourier domain ballistic photons are considered by an infinitely wide Gaussian function or a constant offset. By use of the power series representation of the exponential function the object transfer function can be rewritten as follows:


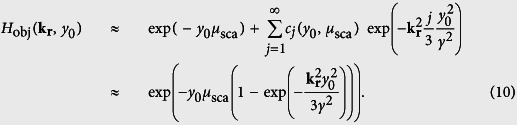


Eq. (10) reveals that the frequency transfer of the fluorescent photons consists of a constant level 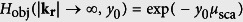 describing the ballistic photons and a Gaussian part describing the diffuse photons. Due to the transfer function’s constant levels 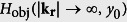 for every plane y_0_, the influence of the object on the detection process can be eliminated by a deconvolution with *H*_obj_(**k**_**r**_, *y*_0_) as long as the intensity of the ballistic photons does not drop below the noise level.

### Contrast coefficient

The detection depth dependent contrast is analyzed by a method derived from the “useful contrast” introduced by Truong *et al.*^32^. The contrast coefficient *Q* is defined by the ration of high and low frequency contents in the image. Therefor xz-slices of the image *p*(*x*, *y*_0_, *z*) are Fourier transformed in x- and z-direction. In Fourier space the image is divided in an image containing the high spatial frequencies (HSF; 
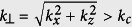
) and one consisting of the low spatial frequencies (LSF; 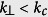). If *k*_*c*_ is chosen in a way that the HSF image contains all useful information of the object and the LSF image is mainly defined by the background signal the contrast coefficient is an appropriate measure of the image contrast.

In contrast to the method introduced by Truong, no upper frequency limit for the useful signal is defined. By the upper limit high frequency noise is separated from the useful signal. If one wants to perform a comparison of different detection depths, this limit is hard to define, since for medium frequencies the signal to noise ratio drops down with increasing detection depth. An alternative is noise adaption. Hereby the Fourier transformations in in x- and z-direction 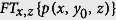 are first normalized by the DC value and white noise is added until the integral 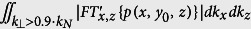 reaches a predefined value *n*_*l*_. If the noise in the original image is white noise and the lateral frequencies 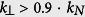 contain no useful information, this procedure leads to images with the same noise level *n*_*l*_ for all detection depths. So a comparison, which is not effected by noise, is possible by the contrast coefficient


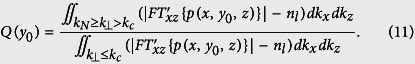


Here 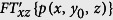 is the Fourier transformation processed by the procedure described above and k_N_ is the Nyquist-frequency.

### Extracting scattering parameters from the image

With the model for *h*_obj_(**r**, *y*_0_) given in eq. (10) the influence of the object itself on the detection is described only by two parameters *μ*_sca_ and *γ*. This method extracts these parameters out of a 3D image stack. Since the most compact form of *h*_obj_(**r**, *y*_0_) is found in Fourier domain, the image stack is first Fourier transformed in x- and z-direction. To eliminate variations of the object spectra the data is smoothed in y-direction. Normalizing the smoothed spectra to a reference spectra at *y*_*0*_ = 0 eliminates the influence of the object spectra on the image process. In this manner the normalized and averaged image spectrum


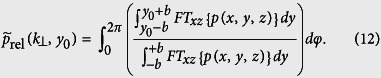


is generated, with 2*b* being the smoothing interval. The integration along the polar angle *φ* (see inset in Fig. 4b)) from 0 to 2π reduces the dimensions of the dataset. So 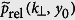 depends on 2 variables *y*_*0*_ and *k*_⊥_. This data is fitted by the model from eq. (10) with *k*_*y*_ = 0 and 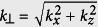. This way the scattering parameters *μ*_sca_ and *γ* are obtained. More detailed information about the theoretical background of this method is given in Supplementary Text 3.

### Depth dependent deconvolution

If the effective illumination *h*_ill_(**r**) and the detection PSF *h*_det_(**r**) as well as the parameters *μ*_sca_ and *γ* describing the influence of the object on the detection are known, the system PSF *h*_sys_(**r**, *y*_0_) can be calculated by eq. (5). As indicated by eq. (6) a perfect depth dependent deconvolution needs a complete 3D deconvolution for every *y*_*0*_. This leads to a huge computation time. Since *h*_sys_(**r**, *y*_0_) is not changing rapidly with *y*_*0*_, a more hardware friendly method is possible. Therefore the 3D image is deconvolved several times by a conventional Wiener filter. For each deconvolution the Wiener filter is optimized for another *y*_*0*_ and each deconvolved image *p*_decon,Wiener_(**r**, *y*_0_) is optimized for one specific *y*_*0*_. The depth dependent deconvolved image *p*_decon_(**r**) is calculated by


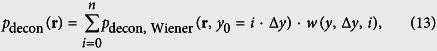


with the window function *w* defined as the shifted Hann window:


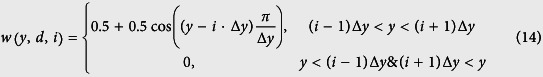


Here 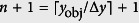 = 12 is the number of sections of thickness Δy and *y*_obj_ is the object dimension in y-direction. The Wiener filter is given by


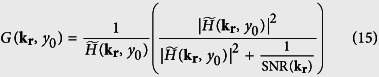


where 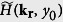 is given by the Fourier transformation of eq. (5). The frequency dependent signal to noise ratio is estimated linearly: 
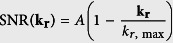
 with 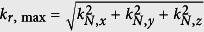 given by the Nyquist frequencies in all directions. The choice of the parameter *A* is a compromise between high frequency enhancement and noise suppression. Models for *h*_ill_(**r**) and *h*_det_(**r**) are obtained by the angular spectrum wave propagation method.

## Additional Information

**How to cite this article**: Meinert, T. *et al.* Separation of ballistic and diffusive fluorescence photons in confocal Light-Sheet Microscopy of Arabidopsis roots. *Sci. Rep.* **6**, 30378; doi: 10.1038/srep30378 (2016).

## Supplementary Material

Supplementary Information

Supplementary Movie 1

Supplementary Movie 2

Supplementary Movie 3

Supplementary Movie 4

## Figures and Tables

**Table 1:** Scattering parameters obtained by fitting the frequency transfer.

	Gauss Conventional	Gauss Confocal	Bessel Conventional	Bessel Confocal
*μ*_sca_ (1/mm)	50	39	42	51
*γ*	22.4	18.9	22.3	15.6
