# Supplementary material for: Separation of ballistic and diffusive fluorescence photons in confocal Light-Sheet Microscopy of Arabidopsis roots
Source: Sci Rep. 2016 Aug 24;6:30378. doi: 10.1038/srep30378 (PMC4995512; doi:10.1038/srep30378)
Supplement: Supplementary Information [file srep30378-s1.pdf]

Supplementary material to
Separation of ballistic and diffusive fluorescence photons in confocal Light-Sheet Microscopy of Arabidopsis roots

Tobias Meinert[1], Olaf Tietz[2], Klaus J. Palme[2], Alexander Rohrbach[1,3]

[1]Laboratory for Bio- and Nano-Photonics, Department of Microsystems Engineering (IMTEK), University of Freiburg, Germany
[2]Institute for Biology II/Botany, Faculty of Biology, University of Freiburg, Germany
[3]BIOSS Centre for Biological Signalling Studies, University of Freiburg, Freiburg, Germany

Supplementary Text1:
yz-slices though unprocessed imaging stacks at different x positions

This section illustrates the influence of the different illumination and detections modes on the image. The quality of the image, mainly defined by resolution and contrast, is determined by the choice of the illumination beam (Gaussian beam or Bessel beam) propagating in the z-direction and by the detection method (widefield or confocal line detection). In addition, the quality decreases strongly with the detection depth y0, because of blur by diffusive fluorescence photons.

This is illustrated in Supplementary Fig. 1 by the yz-slices for 6 different x-positions along the root tip, and for the above-mentioned for different imaging modes.

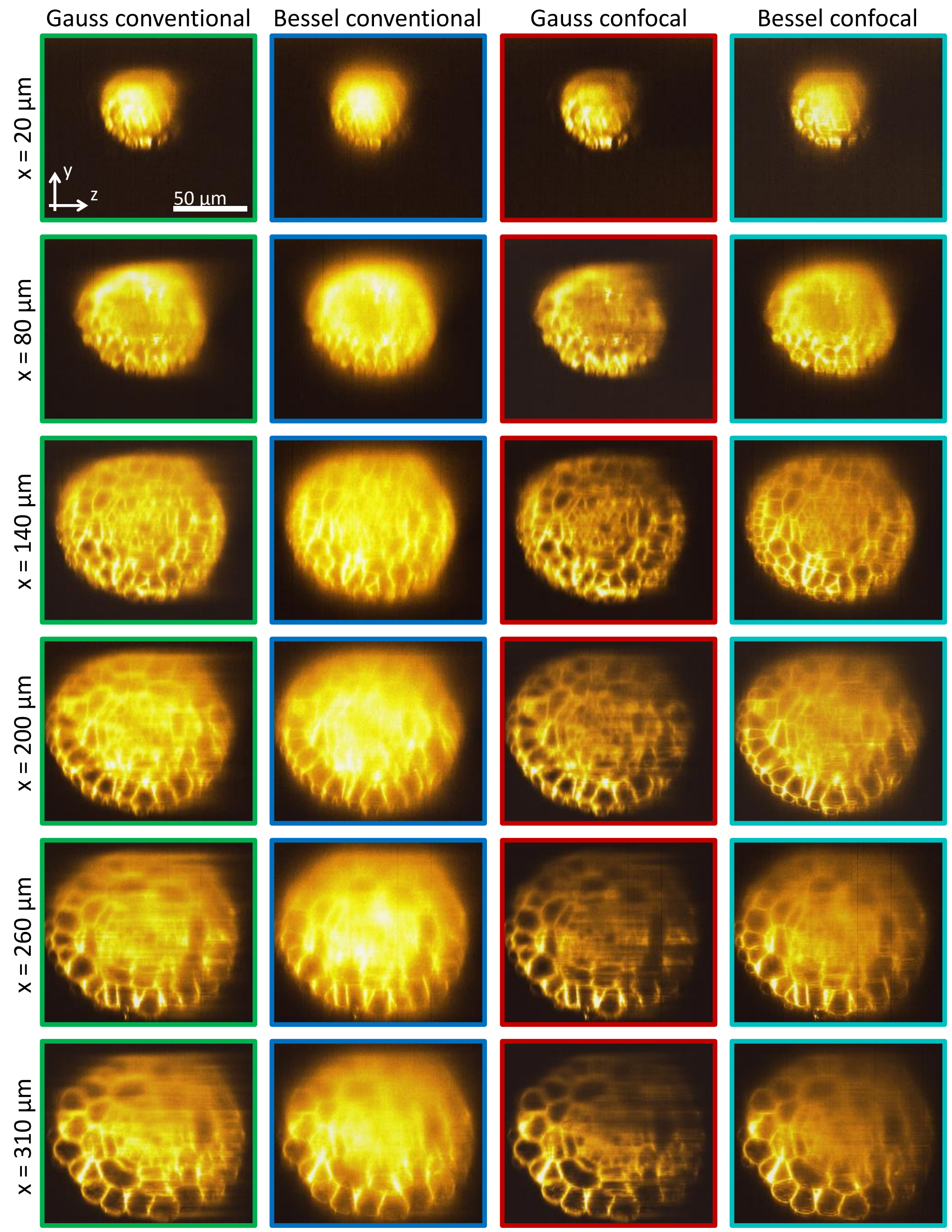

Supplementary Figure 1 | yz-slices through Arabidopsis root imaged by 4 different modes. For each x position Gaussian illumination and especially Bessel illumination with conventional detection results in low contrast. Contrast enhancement by confocal detection is clearly visible. Best contrast is achieved by Gaussian illumination and confocal detection but strong aberrations at the horizontal structures reduce image quality. Bessel Illumination and confocal detection results in good contrast and is nearly free of artefacts.

Supplementary Text2:
On the effect of confocal line detection for Gaussian and Bessel beams

The effect of confocal line detection in light-sheet microscopy (LSM) strongly depends on the illumination beam. This section discusses the difference for Gaussian and Bessel beams.

It is known from theory that confocal line detection in LSM is effective only in detection direction if scattering is not considered (Supplementary Text 6). In LSM with and without confocal detection, the system-PSF is obtained by the multiplication of the detection PSF h_{det} and the effective illumination h_{ill}. If the illumination NA is much smaller than the detection NA, the PSF h_{det} will be much narrower than h_{ill} in x and z direction - even for confocal detection. Consequently confocal detection has mainly an influence on the system PSF, if the profile in detection direction of the light-sheet ($h_{\text{ill}}(\mathbf{r}) \rightarrow \int_{-\infty}^{\infty} h_{\text{SB}}(x - x_s, y, z)\, dx_s$ for conventional detection) and the single beam ($h_{\text{ill}}(\mathbf{r}) \rightarrow h_{\text{SB}}(\mathbf{r})$ for confocal detection) vary. This is not the case for Gaussian illumination, but for Bessel beams.

However Fig. 3 clearly shows that confocal detection improves image contrast for Gaussian illumination as well. This is due to the fact that not only ballistic photons are involved in the image process but also multiple scattered diffusive photons. For diffusive photons the effective detection PSF is much wider as for ballistic photons. So the multiplication with h_{ill} is also effective in x-direction if confocal detection is applied. So we differentiate between the true confocal effect, which describes ballistic photons, and the gating effect by confocal detection, which reduces the influence of diffusive photons.

In Supplementary Fig. 2 the contrast coefficient known from Fig. 3 in the main text is plotted, but this time the two confocal modes are normalized by the corresponding conventional modes. The enhanced contrast improvement for higher detection depth is caused by the gating effect of the confocal slit. In addition Supplementary Fig. 2 shows that contrast improvement for low detection depths is significantly higher for Bessel illumination since gating is less important for low detection depth and the true confocal effect becomes dominant. The later occurs only with Bessel illumination. Regarding to the application in light-sheet microscopy this means that confocal detection in cleared samples where scattering plays a minor role is only beneficial with Bessel illumination. Confocal detection with Gaussian illumination is only useful in scattering samples.

Nevertheless there is a contrast improvement for Gaussian illumination even at low detection depths. This can be explained by scattering of the illumination light, which broadens the illumination beam. The effect of this broadening is minimized by confocal detection.

Both effects true confocal and gating by the confocal slit are also visible by the effective system modulation transfer function (MTF) plotted in Supplementary Fig. 3 and 4. The effective system MTF is given by the Fourier transform of eq. (5). The scattering parameters μ_{sca} and γ were estimated to be 50 mm^{-1} and 22.4 respectively (Realistic data extracted out of the 3D image; see main text). Both Supplementary Fig. 3 and 4 show that for y_0=0 confocal detection expands the MTF for Bessel illumination but has almost no influence on Gaussian illumination. This describes the true confocal effect. As expected, the width of the effective MTF decreases for higher detection depth but confocal detection prevents a fast loss of high frequency information in k_x-direction for Gaussian and Bessel illumination. This describes the gating effect by the confocal slit. Interestingly a similar effect can be observed in k_y-direction for all 4 imaging modes. The gating effect of the confocal slit is theoretically described by the multiplication of the effective illumination beam with the detection PSF. In the confocal case the extent of the effective illumination in x-direction is limited and equal to the extent

in y-direction. Thus the multiplication will truncate the widening of the effective detection PSF in both directions. In x-direction this describes the gating of the confocal slit. In y-direction an inherent property of light-sheet microscopy is described which effects 3D imaging in the same way as the gating effect of confocal detection.

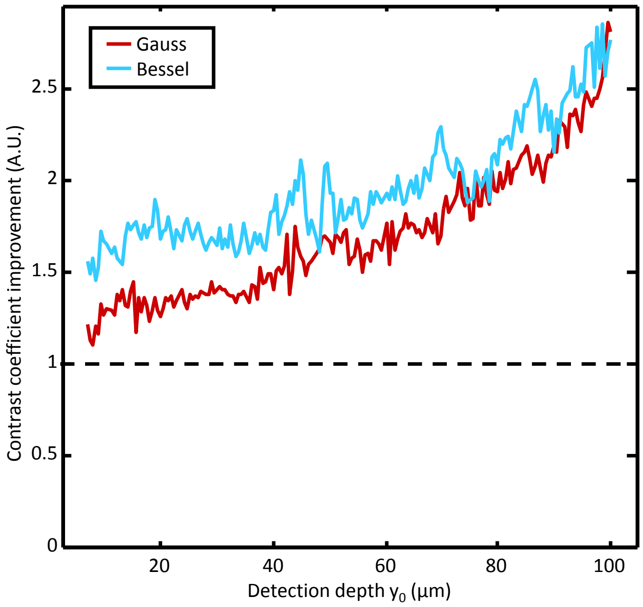

Supplementary Figure 2 | Contrast improvement by confocal detection for Gaussian and Bessel beams. The contrast coefficient of the Gaussian and Bessel confocal mode is normalized by the corresponding conventional modes. Enhanced contrast improvement for high detection depth caused by the gating effect is visible for both types of illumination. For low detection depths contrast improvement is stronger for Bessel illumination since the true confocal effect is dominant.

Fig. 3b exhibits another interesting property of Bessel beam illumination. For the Bessel imaging modes a drop of contrast improvement (compared to the Gaussian conventional mode) is visible for detection depths y_0 between 15µm and 30 µm. With Bessel beam illumination contrast is limited by the fluorescence exited in the ring system, which contributes to the background signal. Due to the cylindrical shape of the object the total amount of fluorophores in the imaging planes changes with the detection depth y_0. For low y_0 the total amount of fluorophores increases in y_0-direction, this increase in turn decreases with y_0. So the ratio of fluorescence emitted by the main lobe and the ring system decreases for higher detection depth, leading to the observed intermediate drop in contrast improvement. This effect is much more pronounced for the Bessel beam since it is much more extended in detection direction than the Gaussian beam. E.g. for the Bessel beam 86% ($1-e^2$) of the photons propagate in a sheet of 30 µm thickness. For the Gaussian Beam this thickness is below 5 µm.

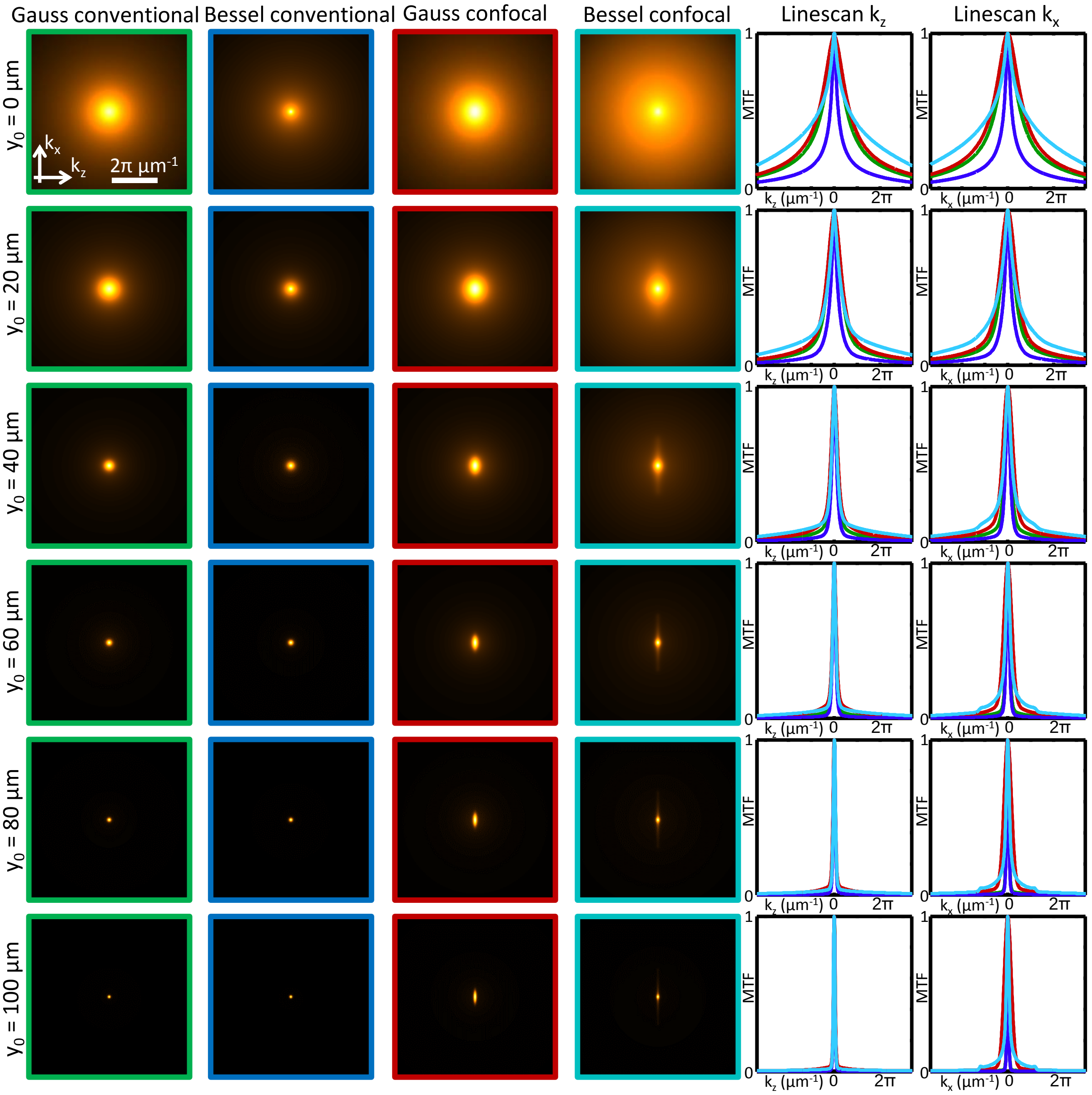

Supplementary Figure 3 | Effective system modulation transfer functions (MTF) $H_{sys}\left(k_x, k_z, y_0\right)$ for varying detection depths *y₀* and different imaging modes. The broadening of the MTF in x-direction by confocal detection is more pronounced for higher detection depth. This is due to the fact that photons displaced by multiple scattering have a higher probability to be blocked by the confocal slit and the image is mainly formed by ballistic photons (gating effect). Further it is shown that without taking account of scattering (y_0 = 0) there is only a negligible image improvement by confocal detection for Gaussian illumination, but a strong improvement for Bessel illumination. With Gaussian illumination contrast improvement is only due to the gating effect.

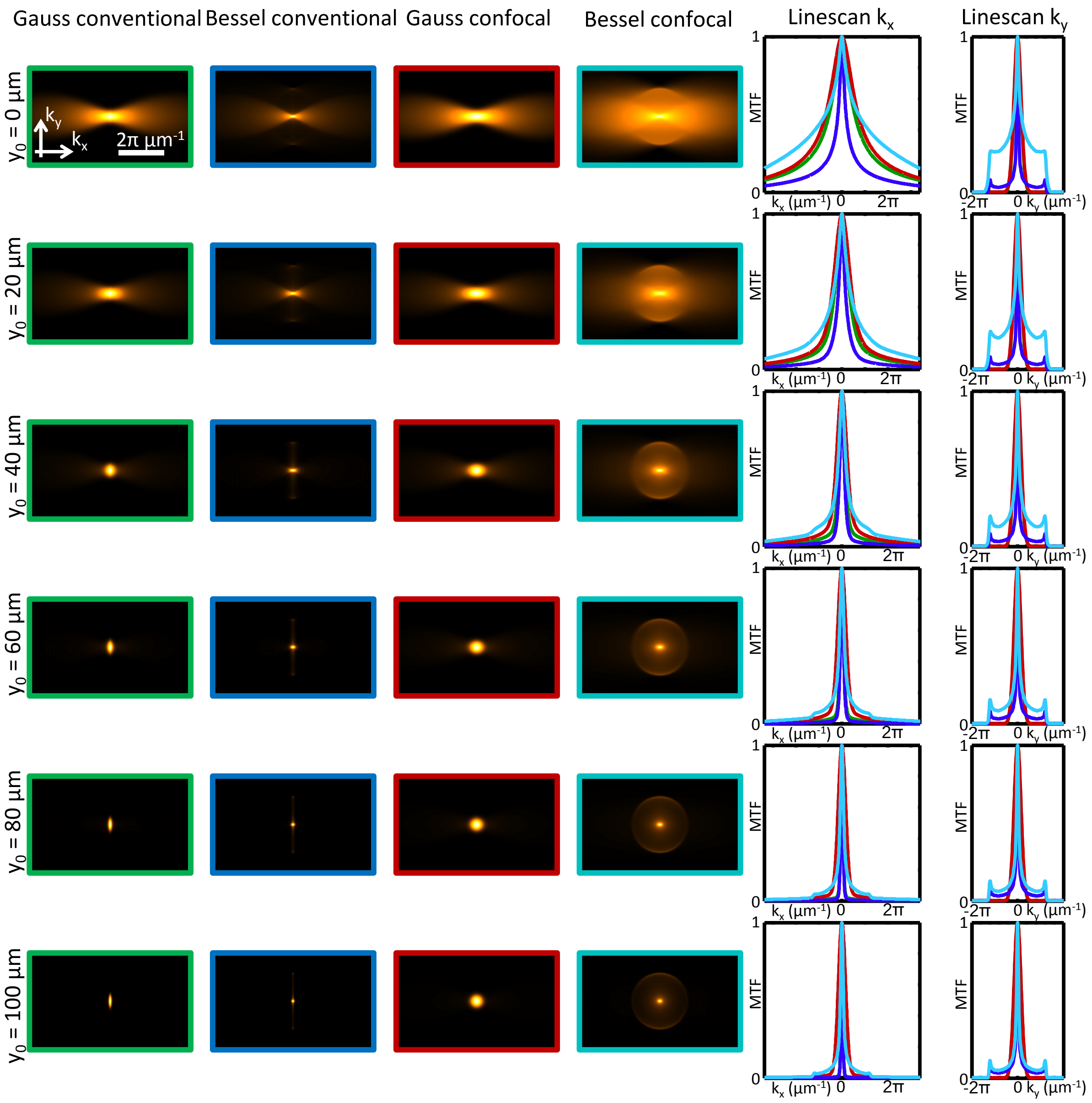

Supplementary Figure 4 | Effective system modulation transfer functions (MTF) $H_{\text{sys}}\left(k_x, k_y, y_0\right)$ **for varying detection depths** $\boldsymbol{y_0}$ **and different imaging modes.** Because of the finite extent of the light-sheet in y-direction the influence of scattering in the detection path is reduced in y-detection direction. This is indicated by the width of the MTF in k_y-direction which is not affected by the detection depth as strongly as the width in k_z-direction (see Supplementary Fig. 3). Mathematically this effect is the same than the gating effect by the confocal slit. This is due to the fact that the effective illumination has the same profile in x- and y-direction for the confocal case. So the multiplication with the effective detection PSF has the same effect in both directions.

Supplementary Text 3:
Theoretical background for the extraction of the scattering parameters μ_{sca} and γ from the 3D image

As described in the main text the scattering parameters μ_{sca} and γ can be extracted by fitting the model given in eq. (10) to the data obtained by the operation described by eq. (12). This section discusses the theoretical background of this procedure.

The general expression for the image spectrum $\int_{y_0-b}^{y_0+b} FT_{xz}\{p(x,y,z)\}dy$ averaged over the distance 2b, describes the numerator as well as the denominator of eq. (12) and is analyzed in detail in the next paragraph.

For a small interval of size 2*b* around y_0 the image $p(x,y,z)$ is given by $h_{sys}(\mathbf{r},y_0)*f(\mathbf{r})$. Due to the Fourier transform in x- and z-direction the convolution converts to a multiplications in these directions but remains a convolution in y-direction:

$$\int_{y_0-b}^{y_0+b} FT_{xz}\{p(x,y,z)\}dy = \int_{y_0-b}^{y_0+b}\int FT_{xz}\{h_{sys}(x,y',z,y_0)\}FT_{xz}\{f(x,y-y',z)\}dy'dy \tag{S1}$$

Since the product $FT_{xz}\{h_{sys}(x,y',z,y_0)\}FT_{xz}\{f(x,y-y',z)\}$ has a finite extent in *y'* the integral region can be reduced to a closed interval and thus the order of integration can be switched:

$$\int_{y_0-b}^{y_0+b} FT_{xz}\{p(x,y,z)\}dy = \int FT_{xz}\{h_{sys}(x,y',z,y_0)\}\int_{y_0-b}^{y_0+b} FT_{xz}\{f(x,y-y',z)\}dydy' \tag{S2}$$

If the object spectra $FT_{xz}\{f(x,y,z)\}$ can be assumed to be approximately constant over 2b, the integral $\int_{y_0-b}^{y_0+b} FT_{xz}\{f(x,y-y',z)\}dy$ is independent of *y'* and can be replaced by a mean object spectrum in k_x- and k_z-direction:

$$\int_{y_0-b}^{y_0+b} FT_{xz}\{p(x,y,z)\}dy \approx \langle FT_{xz}\{f(x,z)\}\rangle \int FT_{xz}\{h_{sys}(x,y',z,y_0)\}dy' \tag{S3}$$

Inserting eq. (S3) in eq. (13) results in

$$\tilde{p}_{rel}(k_\perp,y_0) \approx \int_0^{2\pi} \frac{\int FT_{xz}\{h_{sys}(x,y,z,y_0)\}dy}{\int FT_{xz}\{h_{sys}(x,y,z,y_0=0)\}dy} d\varphi \tag{S4}$$

and shows that the dataset $\tilde{p}_{rel}(k_\perp,y_0)$ is independent of the object. Since the dimensions of the characteristic structures of objects like Arabidopsis root tip vary with y the approximation of eq. (S3) could be critical. This has to be discussed when the dataset is analyzed. The integration along φ can be neglected since $h_{sys}(x,y,z,y_0)$ is rotationally symmetric around the y-axis for conventional detection.

For a deeper understanding of eq. (S4) the term $\int FT_{xz}\left\{h_{\text{sys}}\left(x,\text{y},z,y_0\right)\right\}dy$ has to be discussed in more detail. The system PSF $h_{\text{sys}}\left(x,\text{y},z,y_0\right)$ is given by eq. (5). For ease of calculation, the case of conventional detection is considered where the effective illumination h_{ill} is only a function of y and the Fourier transform in x and z effects solely the convolution of h_{det} and h_{obj}:

$$\int FT_{xz}\left\{h_{\text{sys}}\left(x,\text{y},z,y_0\right)\right\}dy=\int h_{\text{ill}}\left(y\right)\int FT_{xz}\left\{h_{\text{det}}\left(x,\text{y}',z\right)\right\}FT_{xz}\left\{h_{\text{obj}}\left(x,\text{y}-\text{y}',z,y_0\right)\right\}dy'dy \quad \text{(S5)}$$

Since h_{obj} is a δ-function for $y_0 = 0$ eq. (S4) reads

$$\tilde{p}_{\text{rel}}\left(k_\perp,y_0\right)\approx\frac{\int h_{\text{ill}}\left(y\right)\int FT_{xz}\left\{h_{\text{det}}\left(x,\text{y}',z\right)\right\}FT_{xz}\left\{h_{\text{obj}}\left(x,\text{y}-\text{y}',z,y_0\right)\right\}dy'dy}{\int h_{\text{ill}}\left(y\right)FT_{xz}\left\{h_{\text{det}}\left(x,\text{y},z\right)\right\}dy}. \quad \text{(S6)}$$

By analytical methods it can be shown that eq. (S6) is well approximated by eq. (10) from the main text with $k_y=0$ and $k_\perp=\sqrt{k_x^2+k_z^2}$. For computation of the data, $h_{\text{obj}}\left(\mathbf{r}\right)$ has been modeled as a Gaussian beam with NA_{det} = 0.8 and the refractive index of water (1.33). $h_{\text{ill}}\left(y\right)$ has been given by $\exp\left(-\frac{4y^2\,\text{NA}_{\text{ill}}^2\,\pi^2}{\lambda^2}\right)$ with NA = 0.026. For simplification the wavelength λ has been set to 491.5 nm for both detection and illumination. The parameters μ_{sca} and γ have been set to 50 mm^{-1} and 22.4, respectively. The difference between eq. (S6) and eq. (10) cost by the approximation done in eq. (S20) from Supplementary Text 8 is partly compensated. So the root mean square error was below 0.027 for the interval $y_0\in\left[0\ \mu\text{m},\ 100\ \mu\text{m}\right]$ and $k_r\in\left[0\ \mu\text{m}^{-1},\ 2\pi\ \mu\text{m}^{-1}\right]$. As a rule of thumb the error drops down for lower values of NA_{det}.

The same has been done by numerical methods for Bessel beam illumination $\left(h_{\text{SB}}\left(x,y\right)=\text{J}_0\left(\frac{2\pi}{\lambda}\text{NA}\cdot\sqrt{x^2+y^2}\right)\right)$. The step size of k_r was set to $0.02\cdot2\pi$ µm^{-1}. The root mean square error was below 0.0064 and is mainly caused by inaccuracies in the numerical calculations.

Things become more complicate if confocal detection is considered and $h_{\text{ill}}=h_{\text{SB}}$ depends on both x and y. Therefor one expects the dataset to show different behavior in k_x- and k_z-direction. So the integration along φ is critical and should be avoided. It can be shown that the behavior in k_z-direction (k_x=0) can be approximated by a modified version of eq. (10). The gating effect of confocal detection reduces the influence of higher scattering orders on the image formation. The modified version of eq. (10) has to take this into account. It can be shown that for Gaussian illumination the reduced influence of the *j* scattering order on the image formation can be described by the factor $\left(1+j\frac{4\pi^2\,\text{NA}_{\text{ill}}^2\,y_0^2}{3\gamma^2\lambda^2}\right)^{-1/2}$. So the fit function is given by

$$H_{\text{obj}}\left(k_z,y_0\right)=\sum_{j=0}^{\infty}c_j\left(y_0,\mu_{\text{sca}}\right)\left(1+j\frac{16\pi^2\,\text{NA}_{\text{ill}}^2\,y_0^2}{3\gamma^2\lambda^2}\right)^{-1/2}\exp\left(-\mathbf{k}_z^2\,\frac{j}{3}\frac{y_0^2}{\gamma^2}\right). \quad \text{(S7)}$$

With the same values for NA_{ill}, NA_{det}, y_0, λ, γ and μ_{sca} as in the conventional cases and $h_{\text{SB}}\left(x,y\right)=\exp\left(-\frac{4\left(x^2+y^2\right)\text{NA}_{\text{ill}}^2\,\pi^2}{\lambda^2}\right)$ the root mean square error for the gauss conventional mode is below 0.007. (Data has been evaluated in the interval $y_0\in\left[0\ \mu\text{m},\ 100\ \mu\text{m}\right]$ and $k_r\in\left[0\ \mu\text{m}^{-1},\ 2\pi\ \mu\text{m}^{-1}\right]$)

Fitting the models from eq. (10) and eq. (S7) to the frequency transfer (with integration along φ for conventional detection and without for confocal detection (evaluation only along k_z with k_x=0)) of the corresponding imaging modes, one obtains the scattering parameters of Supplementary Table 1.

It is recognized that best fit results are reached for the conventional Gaussian mode since it gives the best signal to noise ratio (SNR) in the frequency transfer. The Bessel conventional mode suffers from low SNR because of its low contrast. The frequency transfer of the confocal modes offer low SNR since they are only evaluated in k_z-direction for k_x=0, whereas the frequency transfer of the conventional modes enables an integration from 0 to 2π over the azimuth angle φ in the Fourier plane. So the values for μ_{sca} and γ (50 mm^{-1} and 22.4 respectively) are most accurate, if the frequency transfer is extracted from the conventional Gaussian mode. Therefore this values have been used for the depth dependent deconvolution.

Supplementary Table 1 | Scattering parameters obtained by fitting the frequency transfer

	Gauss Conventional	Gauss Confocal	Bessel Conventional	Bessel Confocal
μ_{sca} (1/mm)	50	39	42	51
γ	22.4	18.9	22.3	15.6

The result of the 2D fits are illustrated in the Supplementary Movies 1 to 4. (movie 1: Gaussian conventional; movie 2: Gaussian confocal; movie 3: Bessel conventional; movie 4: Bessel confocal)

Supplementary Text 4:
Illustration of the effect of depth dependent deconvolution

The modulation transfer function (MTF) describes the frequency dependent transfer of object information. Usually the transfer drops for higher frequencies. Deconvolution compensates for this frequency dependence of the information transfer. Imaging deep inside scattering media is perturbed by scattering and ballistic photons, being able to transfer high frequency information, are subsequently replaced by diffusive photons, which carry only low frequency information. Consequently the shape of the MTF changes with the detection depth. The deconvolution in areas deep inside the object needs stronger enhancement of high frequency image components than the deconvolution at low detection depths. Standard deconvolution does not accounts for this effect.

Supplementary Fig. 5a shows an yz-slice through an unprocessed image of Arabidopsis root. Supplementary Fig. 5b shows the same slice after depth dependent deconvolution and Supplementary Fig. 5c-e show the slice after deconvolution with a PSF optimized for a specific detection depth y_0. Supplementary Fig. 5c, which is deconvolved with a PSF optimized for y_0=0µm (standard deconvolution) gives good results in the lower image part, but does not enhance high frequencies strong enough to optimize image contrast in the middle and at the top of the image. For very high y_0 almost no ballistic photons remain and the high frequency image components drop below the noise level. Since the PSF is optimized for low detection depth, where these components are above the noise level, they are enhanced. This is leading to a very noisy background in the upper part of the image. The opposite case can be observed in Supplementary Fig. 5e. Deconvolution with a PSF optimized for y_0=80µm enhances the medium frequencies. In the upper part of the image these frequencies are strongly reduced by scattering but still above the noise level. In the lower part of the image these frequencies are enhanced to strong, leading to an overcompensation visible by overshooting, where dark regions become bright again. Supplementary Fig. 5d shows the image slice deconvolved with a PSF optimized for y_0=40µm. So only at this detection depth the removal of diffusive photons has been optimized according to our algorithm. At this depth ballistic photons still have a strong influence. Thus even high frequencies are enhanced leading to strong contrast improvement in the middle part. In the upper part medium frequencies are not enhanced strong enough and high frequencies containing only noise in this region are enhanced too strongly. In the lower part of the image moderate overshooting is visible. A comparison between standard deconvolution (Supplementary Fig. 5c), depth dependent deconvolution from Supplementary Fig. 5b and non depth dependent deconvolution from Supplementary Fig. 5d, is done by the line scans in Supplementary Fig. 5f and g. It is shown that that the depth dependent deconvolution generates high quality images with less noise over the complete 3D volume, while standard deconvolution suffers from low SNR especially for high detection depth and the non depth dependent deconvolution optimized for y_0=40µm results in images with a depth dependent image quality.

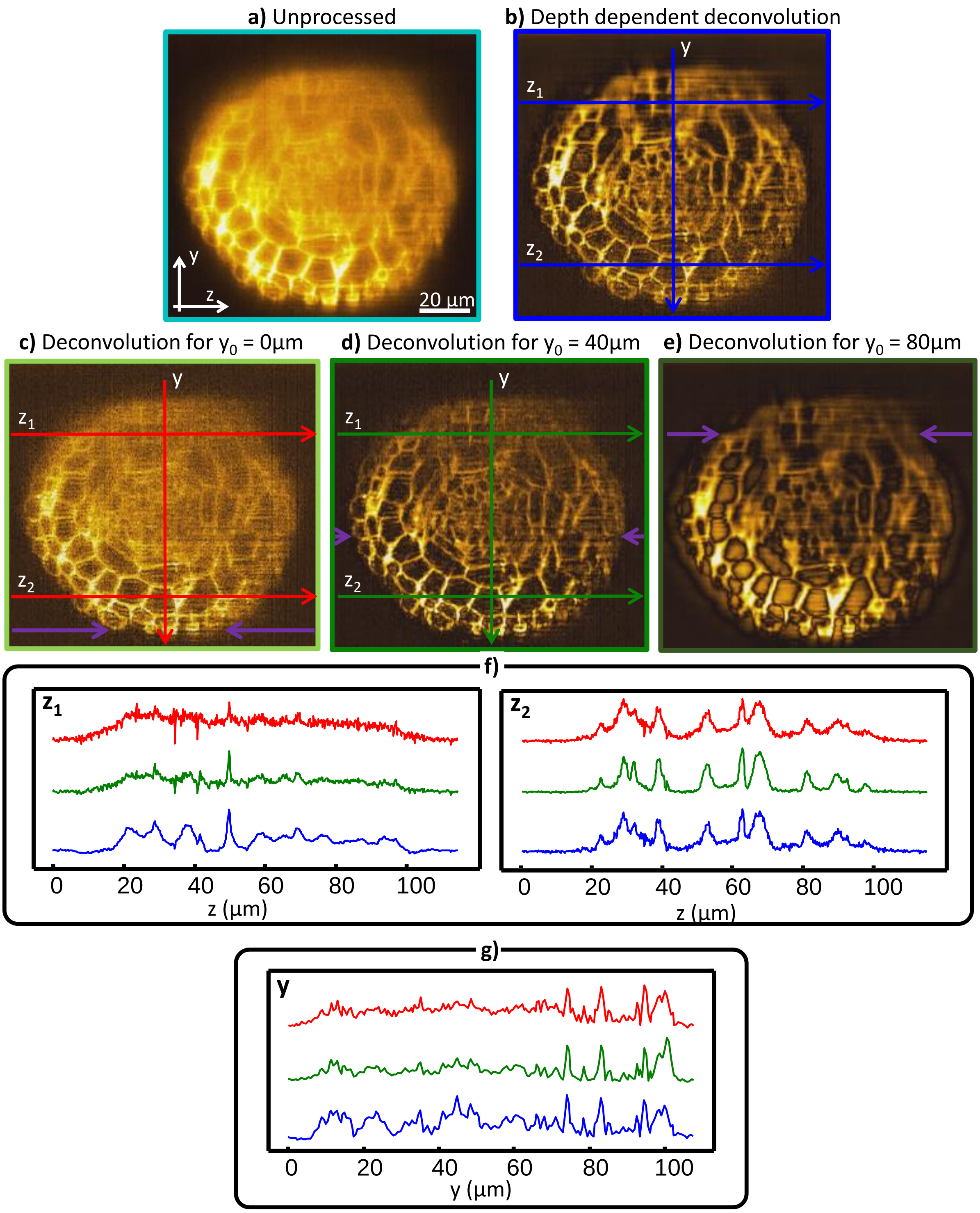

Supplementary Figure 5 | yz-slices through images of Arabidopsis root with different post processing. a) yz-slice through an unprocessed image of Arabidopsis root captured with Bessel beam illumination and confocal detection. **b)** The yz-slice displayed in a) after depth dependent deconvolution. **c)-e)** The yz-slice displayed in a) after deconvolution with a PSF optimized for a specific detection depth (c): y_0=0µm (standard deconvolution); d): y_0=40µm); e): y_0=80µm) **g),f)** Line scans through the image slice after standard deconvolution (red), depth dependent deconvolution (blue) and deconvolution optimized for y_0=40µm (green).

The xz-slices displayed in Supplementary Fig. 6 confirm these findings. The images with depth dependent deconvolution (blue frame) exhibit image contrast, which only slightly depends on the detection depth, while deconvolution optimized for y_0=40µm (dark green frame) results in an image with low contrast and low signal to noise ratio at high detection depth (y_0=70µm). At medium detection

depth (y_0=40µm) image quality is similar to the depth dependent deconvolution, since the deconvolution was optimized for this depth. At low detection depth (y_0=10µm) image contrast is very high, but the insets reveal that this is caused by an overcompensation leading to typical deconvolution aberrations. Standard deconvolution (bright green frame) results in very low SNR for high detection depth.

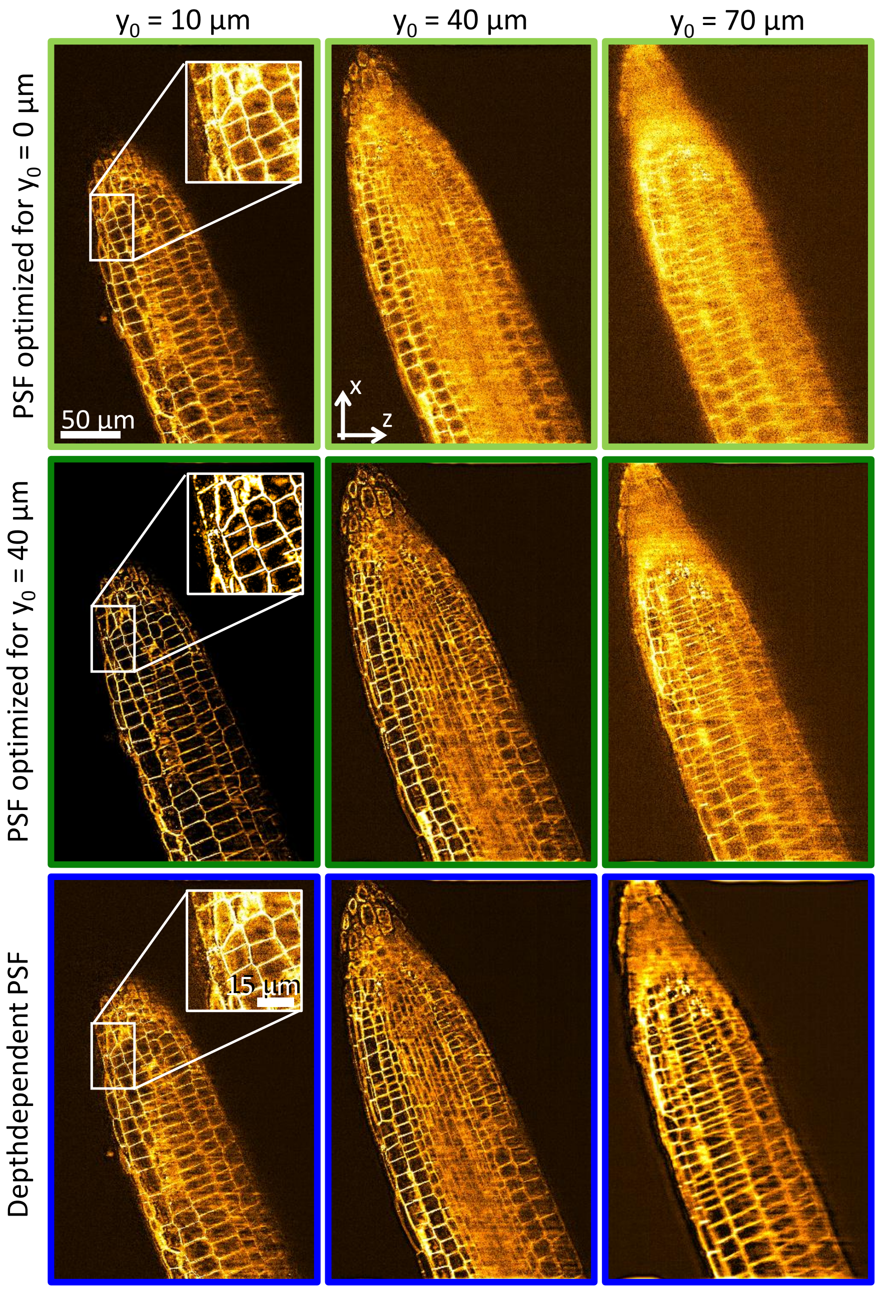

Supplementary Figure 6 | xz-slices through an image of Arabidopsis root with different post processing. xz-slices are shown for different detection depths. In the top row the 3D image was processed by standard deconvolution. In the middle row deconvolution with a PSF optimized for y_0 = 40µm was performed. The bottom row was deconvolved depth dependent. The insets show parts of the image with a scaling of the color bar chosen in a way that bright parts are in saturation. This way typical deconvolution artefacts are observable for the non depth dependent deconvolution optimized for y_0 = 40µm.

Supplementary Text 5:
Depth dependent deconvolution of a bead cluster

As presented in the previous sections depth dependent deconvolution gives very good results when applied to Arabidopsis root tips. However, two simplifications have been made, which are worthy of discussion: First, because of the cylindrical shape of the object the detection depth depends on the lateral image position. Second, the object spectra change with detection depth. To allow a more quantitative characterization of our model the deconvolution has also been tested at an object which do not require these simplifications.

We embedded a mixture of larger fluorescent (Dragon Green) and smaller non-fluorescent polystyrene beads in agarose. The diameter of the fluorescent beads was d_1 = 4.2 µm and the volume concentration was 0.11%. The non-fluorescent beads had a diameter of d_2 = 1.0 µm and a volume concentration of 0.24%. The agarose was formed to a sample with rectangular cross section. For detection, a 20x objective with NA_{det} = 1.0 has been used. The light-sheet was formed by Gaussian or Bessel beams with an axial FWHM of 600 µm. The illumination numerical aperture of the Bessel beam was set to NA_{ill} = 0.15. For confocal line detection the slit width was set to 4 pixels corresponding to $4d_{\text{pixel}} = M_T \cdot 1.3$ µm (with M_T = 20 being the transversal magnification and d_{pixel} = 6.5 µm representing the pixel width). For conventional detection, the slit was set to 154 pixel corresponding to $d_{slit} = M_T \cdot 50$ µm. The sampling in detection direction was chosen to dy = 0.5 µm. 630 layers were imaged leading to a field of view of Δy = 315 µm in detection direction. Each layer has been imaged successively by all 4 imaging modes before switching to the next layer to allow a comparison, which is not affected by drift or bleaching.

The spherical shape of the beads allows a theoretical forecast of the scattering parameters by Mie theory. For the larger beads this predicts a scattering cross section $C_{\text{abs},d1}$ = 25.5 µm². Consequently the scattering coefficient is given by $\mu_{\text{sca},d1} = C_{\text{abs},d1} \cdot \rho_s = 0.76\ \text{mm}^{-1}$ with ρ_s being the density of the larger beads. The scattering anisotropy for the larger beads is given by $g_{\text{HG},d1}$ = 0.92 according to the Henyey-Greenstein (HG) function (see Supplementary Text 8). For the smaller beads, Mie theory predicts $\mu_{\text{sca},d2} = 8.6\ \text{mm}^{-1}$ and $g_{\text{HG},d1}$ = 0.95. The total scattering coefficient is given by $\mu_{\text{sca,tot}} = \mu_{\text{sca},d1} + \mu_{\text{sca},d2}$ = 9.36 and the total anisotropy factor $g_{\text{HG,tot}}$ = 0.95 is given by $g_{\text{HG},d2}$ since $\mu_{\text{sca},d2} >> \mu_{\text{sca},d1}$. The scattering parameters extracted out of the 3D image of the beads are summarized in Supplementary Table 2.

Supplementary Table 2 | Scattering parameters obtained by fitting the frequency transfer of a bead cluster

	Gauss Conventional	Gauss Confocal	Bessel Conventional	Bessel Confocal	Mie theory
μ_{sca} (1/mm)	12.5	15.4	12.7	21.6	9.36
γ	8.99	8.98	8.82	5.52	6.3
g_{HG}	0.975	0.975	0.974	0.934	0.95

The conventional modes give the highest reliability for the fitting algorithm since the integration along the azimuth angle increases the signal to noise ratio. The relatively small deviation of theoretical and extracted scattering coefficient confirms that our model for photon separation is a good description of the highly complex and random processes which occur while imaging in scattering media. Supplementary Fig. 8 shows that the phase function given by Mie theory and the estimated Gaussian phase function (g = 8.99) are in good agreement as well.

The parameters extracted by the Gaussian conventional mode have also been used for depth dependent deconvolution. To avoid the inherent critical estimation of the SNR, Lucy-Richardson deconvolution has been applied. In order to speed up the deconvolution, the window function (see Methods section) was first multiplied to the image and the deconvolution was calculated only for the non-zero area. 10 different PSFs were calculated and 10 deconvolutions were performed. The extend of the convolution area in detection direction was given by $2 \cdot \lceil \Delta y / 9 \rceil$ for the inner layers and by $\lceil \Delta y / 9 \rceil$ for the top most and bottom most layer. The extend of the PSF in detection direction was chosen to be halve of the extend of the convolution area. For the Bessel conventional mode this leads to a slight truncation of the PSF causing a modulation in the final image. This could be avoided in future work by zero padding or choosing larger convolution areas.

Supplementary Fig. 8a-c show images of the larger (d_1 = 4.2 µm) fluorescent beads at different detection depths and formed by different imaging modes. These figures further show the unprocessed images and the images after depth dependent deconvolution and standard deconvolution (section-wise deconvolution as described above but no adaption of the PSF). It can be seen that for all imaging modes and detection depths, the depth dependent deconvolution reduces noise and is able to reconstruct the spherical shape of the beads up to a certain degree. For high detection depths, the non depth dependent deconvolution leads to strong noise enhancement. Because of the low scattering anisotropy of the small beads, diffusive photons even with small scattering orders result in such a wide PSF that they mainly contribute to the background signal. Consequently, a depth dependent deconvolution compensates for this increase in background signal. The autoscaling of small image details makes the effect of background reduction invisible.

Therefore, Supplementary Fig. 8d shows a maximum projection (in x direction for the images and in x and z direction for the line plots) of the beads images with Bessel beam illumination and confocal detection. The original image is compared to images deconvolved with a depth dependent PSF and PSFs optimized for a detection depth of y_0 = 0 µm and y_0 = 144 µm respectively. One can clearly see that deconvolution increases the contrast. However, only our depth dependent deconvolution provides good results also for high detection depths.

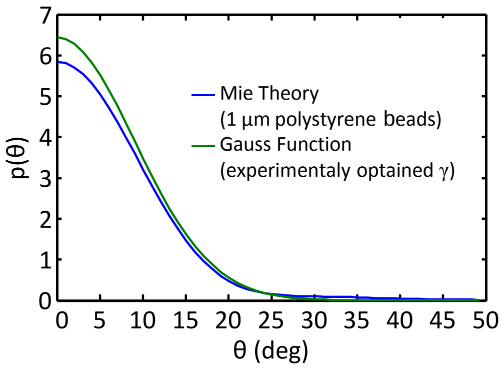

Supplementary Figure 7 | Scattering phase function. Comparison of the phase function given by Mie theory and the estimated Gaussian phase function.

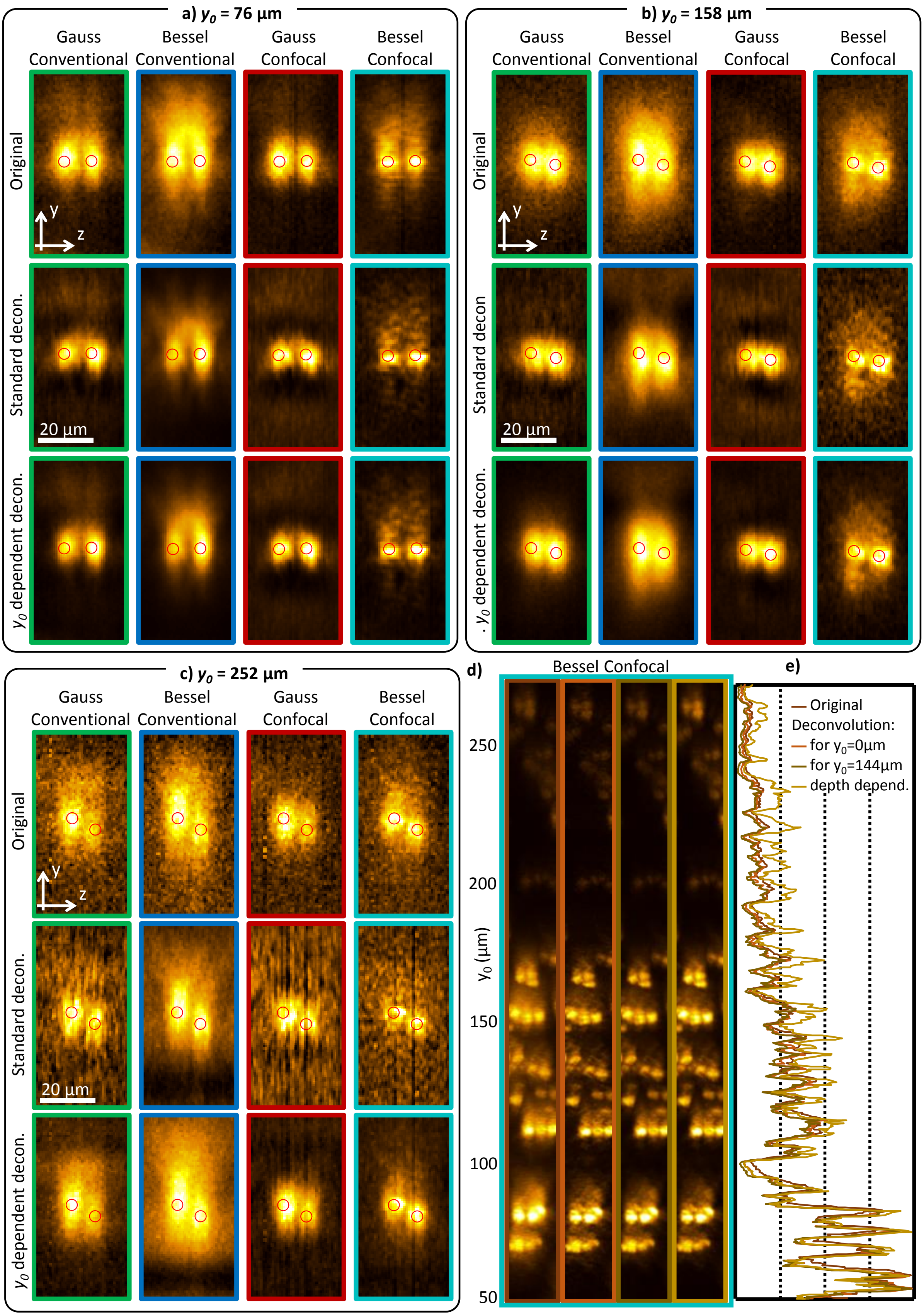

Supplementary Figure 8 | Images of large beads before and after deconvolution. a)-c) images of 4 µm beads without post processing, after depth dependent deconvolution and after standard deconvolution. The detection depth y_0 of the beads in a), b) and c) are 76 µm, 159µm and 252 µm respectively. **d)** Maximum projection in x-direction (images) and in x- and z-direction (line plots, **e)**) of beads imaged by confocal Bessel mode using different post-processing.

Supplementary Text 6:
On the image formation in LSM with static light-sheet and scanned light-sheet with and without confocal line detection

This section discusses the image formation for light-sheet microscopy in a very general way. First, we calculate an effective illumination PSF $h_{\text{ill}}(\mathbf{r})$, seen by one pixel line during its exposure time. Hereby it doesn't matter whether all pixel lines are exposed at the same time or one by one. The first is the case in conventional widefield detection. The latter is given if confocal line detection is realized by the rolling shutter of the camera.

For a static light-sheet the effective illumination is simply given by the light-sheet. For the scanned light-sheet the single illumination beam $h_{\text{SB}}(\mathbf{r})$ is scanned in x-direction with the velocity *v*. So the illumination at time *t* is given by $h_{\text{SB}}(\mathbf{r}-vt\cdot\mathbf{e}_x)$. To find the effective illumination one has to integrate over the exposure time *T* of the pixel line:

$$\begin{aligned} h_{\text{ill}}(\mathbf{r}) &= \int_{-T/2}^{T/2} h_{\text{SB}}(\mathbf{r}-vt\cdot\mathbf{e}_x)\,dt \\ &= \int_{-\infty}^{\infty} h_{\text{SB}}(\mathbf{r}-vt\cdot\mathbf{e}_x)\,\text{rect}\left(\tfrac{t}{T}\right)dt \\ &= \int_{-\infty}^{\infty} h_{\text{SB}}(\mathbf{r}-vt\cdot\mathbf{e}_x)\,\text{rect}\left(\tfrac{vt}{d_{\text{slit}}}\right)dt \\ &= \frac{1}{v}\int_{-\infty}^{\infty} h_{\text{SB}}(\mathbf{r}-x_s\cdot\mathbf{e}_x)\,\text{rect}\left(\tfrac{x_s}{d_{\text{slit}}}\right)dx_s \end{aligned} \tag{S8}$$

Here, the scanning speed has been set to $\frac{d}{dt}x_s = v = -\frac{1}{M}\frac{d_{\text{slit}}}{T}$ with d_{slit} representing the width of the confocal slit, if confocal detection is applied. For better readability the transversal magnification of the detection optics is set to *M* = -1. The last line of eq. (S8) indicates that the effective illumination is given by the convolution of the illumination beam with the scanning trajectory limited to the time interval in which the pixel line of the camera is active. Using a pictorial language one can say that the effective illumination is the result of drawing the scanning trajectory with $h_{\text{SB}}(\mathbf{r})$ as paint-brush. If d_{slit} is very small, the scanning trajectory reduces to a point and $h_{\text{ill}}(\mathbf{r})$ is given by $h_{\text{SB}}(\mathbf{r})$.

The effective illumination excites fluorescence, which is given by $f(\mathbf{r}-y_0\cdot\mathbf{e}_y)\cdot h_{\text{ill}}(\mathbf{r}-x_0\cdot\mathbf{e}_x)$ with $f(\mathbf{r}-y_0\cdot\mathbf{e}_y)$ being the fluorophore density shifted by the detection depth y_0 and $h_{\text{ill}}(\mathbf{r}-x_0\cdot\mathbf{e}_x)$ being the effective illumination for a pixel line at position x_0. (The shift by y_0 indicates the object scanning in y-direction.) The fluorescent signal is imaged by the detection path of the light-sheet microscope. This is described by a convolution with the detection PSF $h_{\text{det}}(\mathbf{r})$:

$$p(\mathbf{r},x_0,y_0) = \left(f(\mathbf{r}-y_0\cdot\mathbf{e}_y)\cdot h_{\text{ill}}(\mathbf{r}-x_0\cdot\mathbf{e}_x)\right) * h_{\text{det}}(\mathbf{r}) \tag{S9}$$

The pixel line captures a line out of the 3D image $p(\mathbf{r})$ at $y=0$ and $x=x_0$. The latter is only true if the beam scanning is synchronized to the exposure of the pixel lines, like it is the case for confocal detection. But this assumption has also no effect for conventional detection since the effective illumination is assumed to be independent of *x* in this case. So for all cases the final image is given by

$$\begin{aligned} p(x_0,y_0,z) &= \iint \left(f\left(\mathbf{r}-y_0\cdot\mathbf{e}_y\right) h_{\text{ill}}\left(\mathbf{r}-x_0\cdot\mathbf{e}_x\right)\right) * h_{\text{det}}(\mathbf{r})\delta(y)\delta(x-x_0)\,dxdy \\ &= \iint \iiint \left(f\left(\mathbf{r}'-y_0\cdot\mathbf{e}_y\right) h_{\text{ill}}\left(\mathbf{r}'-x_0\cdot\mathbf{e}_x\right)\right)\cdot h_{\text{det}}(\mathbf{r}-\mathbf{r}')d\mathbf{r}'\delta(y)\delta(x-x_0)\,dxdy \,. \qquad \text{(S10)} \\ &= \iiint f(x',y'-y_0,z') h_{\text{ill}}(x'-x_0,y',z')\cdot h_{\text{det}}(x_0-x',0-y',z-z')dx'dy'dz' \end{aligned}$$

The difference between confocal and conventional detection is described by $h_{\text{ill}}(\mathbf{r})$, which is the single beam in the confocal case and the x-independent light-sheet in the conventional case. The last line of eq. (S10) represents a conventional image $(f\cdot h_{\text{ill}})*h_{\text{det}}$ in z-direction and a confocal image $(h_{\text{det}}\cdot h_{\text{ill}})*f$ in y-direction. In x-direction, the image is a confocal one, if $h_{\text{ill}}(\mathbf{r})$ depends on x (confocal detection) and the image is a conventional one, if $h_{\text{ill}}(\mathbf{r})$ does not depend on x (conventional detection).

To find an expression for the system PSF $h_{\text{sys}}(\mathbf{r})$ the object $f(\mathbf{r})$ has to be assumed as point object at $r_p=(x_p,y_p,z_p)$:

$$\begin{aligned} p_{\text{point}}(x_0,y_0,z,\mathbf{r_p}) &= \iiint \delta(x'-x_p,y'-y_p-y_0,z'-z_p) h_{\text{ill}}(x'-x_0,y',z')\cdot h_{\text{det}}(x_0-x',-y',z-z')dx'dy'dz' \\ &= h_{\text{ill}}(x_s-x_p,y_0+y_p,z_p)\cdot h_{\text{det}}(x_0-x_p,y_0+y_p,z-z_p) \end{aligned}$$

(S11)

Since $h_{\text{ill}}(\mathbf{r})$ is independent on z the system PSF can be written as $h_{\text{sys}}(\mathbf{r})=h_{\text{ill}}(\mathbf{r})h_{\text{det}}(\mathbf{r})$. It is important to mention that the multiplication of $h_{\text{det}}(\mathbf{r})$ with $h_{\text{ill}}(\mathbf{r})$ has only an effect in directions in which $h_{\text{ill}}(\mathbf{r})$ is not constant. Of course there is an effect in y-direction. This expresses the sectioning capability of light-sheet microscopy. There is no effect in z-direction and there is only an effect in x-direction if confocal line detection is applied.

Since even for confocal detection $h_{\text{det}}(\mathbf{r})$ is much thinner than $h_{\text{ill}}(\mathbf{r})$ in x-direction, the effect in x-direction is very week. Consequently confocal detection only increases contrast if $h_{\text{ill}}(\mathbf{r})$ for conventional detection compared to $h_{\text{ill}}(\mathbf{r})$ for confocal detection is not only extended in x-direction, but also in y-direction. This is the case for Bessel beams, but not for Gaussian beams. So the true confocal effect is only given for Bessel beam illumination. Contrast improvement by confocal detection with Gaussian illumination appears only in scattering objects.

Supplementary Text 7:
On the derivation of the distribution of fluorescent photons over the scattering orders

This section discusses the probability for a fluorescent photon to be scattered *j*-times on its way from the focal plane to the detection objective. If a fluorescent photon is emitted at the focal plane it has to travel a distance given by the detection depth y_0 in y´ direction through the object to reach the objective (see Fig. 4b from the main text). (For simplification the cylindrical shape of the object is neglected.) If the probability to be scattered while traveling a certain distance in *y´* direction is given by μ_{sca} the following set of differential equations can be formulated:

$$\begin{aligned} \frac{\partial c_0(y')}{\partial y'} &= -\mu_{\text{sca}} \cdot c_0(y') \\ \frac{\partial c_j(y')}{\partial y'} &= -\mu_{\text{sca}} \cdot c_j(y') + \mu_{\text{sca}} \cdot c_{j-1}(y') \quad \text{for} \quad j > 0 \end{aligned} \tag{S12}$$

Hereby $c_j(y')$ is the percentage of *j*-times scattered photons at a distance *y´* from the focal plane. The assumption that at the focal plane all emitted photons are unscattered $\left(c_0(y'=0)=1;\, c_{j>0}(y'=0)=0\right)$ results in

$$c_j(y_0, \mu_{\text{sca}}) = \tfrac{1}{j!} \cdot (\mu_{\text{sca}} y_0)^j \cdot \exp(-\mu_{\text{sca}} y_0)\,. \tag{S13}$$

Eq. (S13) describes the percentage of *j* times scattered photons involved in the image formation for a detection depth of y_0.

Supplementary Text 8:
On the derivation of the scattering order specific object PSF $h_{\mathrm{obj},j}(\mathbf{r},\mathrm{y}_0,\gamma)$

This section discusses how the influence of the object onto the detection process can be modeled specifically for each scattering order.

For ballistic photons (zero scattering order) the detection process is not influenced by the object. Thus $h_{\mathrm{obj},0}(\mathbf{r})$ is given by the Dirac delta-function $\delta(\mathbf{r})$.

A scattering event changes the propagation angle of the photon. In this article a new scattering phase function is introduced in order to enable a compact description of the influence of scattering on the image process. The phase function is based on a Gaussian distribution and thus has an analytical solution in Fourier space. The introduced model assumes that the probability density function (PDF) for the scattering angle $\mathrm{PDF}_\theta\left(\sin\left(\frac{\theta}{2}\right)\right) \approx \mathrm{PDF}_\theta\left(\frac{\lambda}{4\pi}\cdot \mathrm{k}_\perp\right)$ is given by a normal distributed with a variance of $\frac{1}{\gamma^2}$:

$$\mathrm{PDF}_\theta(\theta,\gamma) = \frac{\gamma^2}{\left(1-e^{-\gamma^2}\right)4\pi}\exp\left(-\left(\sin\left(\frac{\theta}{2}\right)\right)^2\gamma^2\right) \tag{S14}$$

A scattering phase function has to fulfill 2 conditions. First the integral over the 4π space has to be 1: $2\pi\int_0^\pi P(\theta)\sin(\theta)d\theta = 1$. This condition is fulfilled by choosing a proper prefactor. Second the scattering anisotropy $\langle\cos(\theta)\rangle = 2\pi\int_0^\pi P(\theta)\cos(\theta)\sin(\theta)d\theta$ has to be given by the anisotropy factor g_{HG} known e.g. from the Henyey-Greenstein function. This results in the relation $g_{\mathrm{HG}} = \coth\left(\frac{\gamma^2}{2}\right) - \frac{2}{\gamma^2}$ with coth being the hyperbolic cotangent.

The influence on the detection process introduced by a change of the propagation angle depends on the distance of the scattering event from the focal plane e.g. a scattering event in close proximity to the focal plane does not affect the image formation. As a first approximation, the displacement Δr (see Fig. 4b in the main text) of one photon in the 3D image caused by a change of the propagation angle by θ depends linearly on the distance of the scattering event from the focal plane y_{sca}: $\Delta r = \sin(\theta)y_{\mathrm{sca}} \approx 2\sin\left(\frac{\theta}{2}\right)y_{\mathrm{sca}}$

Hence, the PDF for photon displacements caused by single scattering events $\mathrm{PDF}_{\mathrm{single},\Delta\mathbf{r}}(\Delta\mathbf{r})$, is given by

$$\mathrm{PDF}_{\mathrm{single},\Delta\mathbf{r}}(\Delta\mathbf{r},y_{\mathrm{sca}},\gamma) = \frac{\gamma^3}{\left(2\sqrt{\pi}y_{\mathrm{sca}}\right)^3}\exp\left(\frac{-\Delta\mathbf{r}^2\gamma^2}{4y_{\mathrm{sca}}^2}\right). \tag{S15}$$

If only photons of the first scattering order are considered, the mean scattering position $\langle y_{\mathrm{sca}}\rangle$ is given by half of the detection depth $\frac{y_0}{2}$ and $h_{\mathrm{obj},1}(\mathbf{r})$ is given by the PDF for photon displacement caused by a single scattering event at $\frac{y_0}{2}$ (prefactor is neglected for simplicity):

$$h_{\text{obj},1}(\mathbf{r}, y_0, \gamma) = \exp\left(\frac{-\Delta\mathbf{r}^2\gamma^2}{y_0^2}\right). \quad \text{(S16)}$$

If one photon undergoes a second scattering event, the PDF for photon displacements is further widened. Thus, for multiple scattering events the PDF is given by the convolution between PDFs for single scattering events:

$$\text{PDF}_{\text{multi},\Delta\mathbf{r}}(\Delta\mathbf{r}) = \text{PDF}_{\text{single},\Delta\mathbf{r}}(\Delta\mathbf{r}, y_{\text{sca},1}) * \text{PDF}_{\text{single},\Delta\mathbf{r}}(\Delta\mathbf{r}, y_{\text{sca},2}) * \ldots * \text{PDF}_{\text{single},\Delta\mathbf{r}}(\Delta\mathbf{r}, y_{\text{sca,i}}) * \ldots \quad \text{(S17)}$$

If only those photons are considered that have been scattered j times (*j*th scattering order), the *i*th scattering event ($i < j+1$) of the photons takes place at a mean distance $\bar{y}_{\text{j,i}} = \frac{i}{j+1} y_0$ from the focal plane and $h_{\text{obj,j}}(\mathbf{r})$ is given by (prefactor are neglected for simplicity)

$$h_{\text{obj,j}}(\mathbf{r}, y_0, \gamma) = \exp\left(-\frac{\mathbf{r}^2\gamma^2}{\left(2\frac{1}{j+1}y_0\right)^2}\right) * \exp\left(-\frac{\mathbf{r}^2\gamma^2}{\left(2\frac{2}{j+1}y_0\right)^2}\right) * \ldots * \exp\left(-\frac{\mathbf{r}^2\gamma^2}{\left(2\frac{i}{j+1}y_0\right)^2}\right). \quad \text{(S18)}$$

A derivation for $\bar{y}_{\text{j,i}}$ can be found in Supplementary Text 9.

In Fourier domain one finds a more compact expression:

$$\begin{aligned} H_{\text{obj,j}}(\mathbf{k_r}, y_0, \gamma) &= \prod_{i=1}^{j} \exp\left(-\mathbf{k}_\mathbf{r}^2 \left(\frac{\frac{i}{j+1}y_0}{\gamma}\right)^2\right) \\ &= \exp\left(-\mathbf{k}_\mathbf{r}^2 \frac{j(1+2j)}{6(1+j)} \frac{y_0^2}{\gamma^2}\right) \end{aligned}. \quad \text{(S19)}$$

With the approximation $\frac{j(1+2j)}{6(1+j)} \approx \frac{j}{3}$ this can be simplified to

$$H_{\text{obj,j}}(\mathbf{k_r}, y_0, \gamma) \approx \exp\left(-\frac{1}{3}\mathbf{k}_\mathbf{r}^2 \left(\sqrt{j}\frac{y_0}{\gamma}\right)^2\right) \quad \text{(S20)}$$

and the invers Fourier transform result in (prefactor is neglected for simplicity)

$$h_{\text{obj,j}}(\mathbf{r}, y_0, \gamma) \approx \exp\left(-\frac{3\mathbf{r}^2\gamma^2}{4\left(\sqrt{j}y_0\right)^2}\right). \quad \text{(S21)}$$

It should be mentioned that $H_{\text{obj,j}}(\mathbf{k_r} = 0)$ in eq. (S19) and (S20) is equal to 1. Consequently, no prefactor is needed to scale the integral over the whole 3D real space to 1 in order to fulfill the basic requirement of PDFs.

Supplementary Fig. 9 shows the difference of $H_{\text{obj}}(\mathbf{k_r})$ (calculated as described in the main text; μ_{sca} and γ have been set to 40 mm^{-1} and 21 respectively) with and without the approximation of eq. (S20). If the model with approximation is used as fit function, it will result in higher values for γ. Since in the framework of this article the physical meaning of this value is of no further interest the approximation is an appropriate method to simplify the model and helps do understand the physical basics of image formation under scattering conditions.

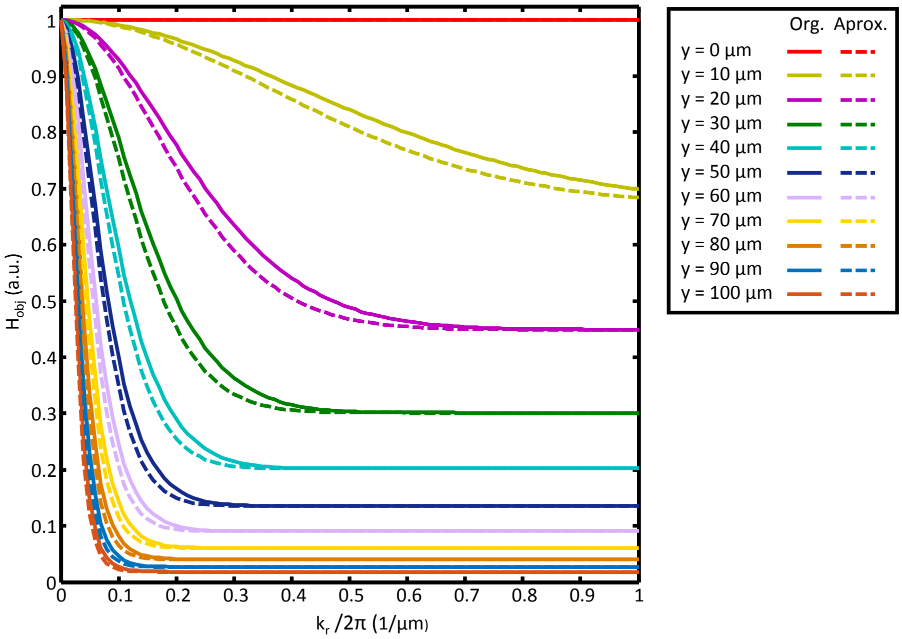

Supplementary Figure 9 | Comparison of $H_{\text{obj}}(\mathbf{k_r})$ **with and without approximation.**

Supplementary Text 9: On the derivation of $\overline{y}_{i,j}$

The distance to the *i*th scattering event to the focal plane depends on the scattering order of the photon after it has left the object. This distance is critical for the width of the scattering order dependent object PSF and is discussed in this section.

The probability for the *i*th scattering event is proportional to $\mu_{\text{sca}} c_{i-1}(y')$. The probability that this scattered photon belongs to the photon group c_j after it has left the object, is given by $c_{j-i}(y_0 - y')$. So the mean distance to the focal plane of the *i*th scattering event of a photon that belongs to the group c_j is given by

$$\overline{y}_{j,i} = \frac{\int_0^{y_0} y' \mu_{\text{sca}} c_{i-1}(y') c_{j-i}(y_0 - y') dy'}{\int_0^{y_0} \mu_{\text{sca}} c_{i-1}(y') c_{j-i}(y_0 - y') dy'} . \quad \text{(S22)}$$

For $i < j+1$ (a natural condition for the above described process) eq. (S22) can be simplified to

$$\overline{y}_{j,i} = y_0 \frac{\Gamma(1+i) \cdot \Gamma(1+j)}{\Gamma(i) \cdot \Gamma(2+j)} = y_0 \frac{i}{j+1} . \quad \text{(S23)}$$

Here, Γ is the gamma function.

Supplementary Video 1: Fitting the frequency transfer through an Arabidopsis root tip imaged with Gaussian Illumination and conventional detection

The frequency transfer through an Arabidopsis root tip is fitted by the scattering model introduced in this article. The frequency transfer depends on the detection depth y_0 and the spatial frequency $k_\perp$. The fitting parameters are the scattering coefficient μ_{sca}, and γ describing the scattering anisotropy. The video shows the fit for the 3D image captured with Gaussian illumination and conventional detection.

Supplementary Video 2: Fitting the frequency transfer through an Arabidopsis root tip imaged with Gaussian Illumination and confocal detection

The frequency transfer through an Arabidopsis root tip is fitted by the scattering model introduced in this article. The frequency transfer depends on the detection depth y_0 and the spatial frequency $k_\perp$. The fitting parameters are the scattering coefficient μ_{sca}, and γ describing the scattering anisotropy. The video shows the fit for the 3D image captured with Gaussian illumination and confocal detection.

Supplementary Video 3: Fitting the frequency transfer through an Arabidopsis root tip imaged with Bessel Illumination and conventional detection

The frequency transfer through an Arabidopsis root tip is fitted by the scattering model introduced in this article. The frequency transfer depends on the detection depth y_0 and the spatial frequency $k_\perp$. The fitting parameters are the scattering coefficient μ_{sca}, and γ describing the scattering anisotropy. The video shows the fit for the 3D image captured with Bessel illumination and conventional detection.

Supplementary Video 4: Fitting the frequency transfer through an Arabidopsis root tip imaged with Bessel Illumination and confocal detection

The frequency transfer through an Arabidopsis root tip is fitted by the scattering model introduced in this article. The frequency transfer depends on the detection depth y_0 and the spatial frequency $k_\perp$. The fitting parameters are the scattering coefficient μ_{sca}, and γ describing the scattering anisotropy. The video shows the fit for the 3D image captured with Bessel illumination and confocal detection.
